# Molecular insights into antibiotic resistance - how a binding protein traps albicidin

**DOI:** 10.1038/s41467-018-05551-4

**Published:** 2018-08-06

**Authors:** Lida Rostock, Ronja Driller, Stefan Grätz, Dennis Kerwat, Leonard von Eckardstein, Daniel Petras, Maria Kunert, Claudia Alings, Franz-Josef Schmitt, Thomas Friedrich, Markus C. Wahl, Bernhard Loll, Andi Mainz, Roderich D. Süssmuth

**Affiliations:** 1 Institut für Chemie, Biologische Chemie, Technische Universität Berlin, Straße des 17. Juni 124, 10623 Berlin, Germany; 2 Institut für Chemie und Biochemie, Strukturbiochemie, Freie Universität Berlin, Takustraße 6, 14195 Berlin, Germany; 3 Skaggs School of Pharmacy & Pharmaceutical Sciences, University of California San Diego, 9500 Gilman Drive, La Jolla, CA 92093-0751 USA; 4 Institut für Chemie, Biophysikalische Chemie, Technische Universität Berlin, Straße des 17. Juni 135, 10623 Berlin, Germany; 5 Helmholtz Zentrum Berlin für Materialien und Energie, Macromolecular Crystallography, Albert-Einstein-Straße 15, D-12489 Berlin, Germany

## Abstract

The worldwide emergence of antibiotic resistance poses a serious threat to human health. A molecular understanding of resistance strategies employed by bacteria is obligatory to generate less-susceptible antibiotics. Albicidin is a highly potent antibacterial compound synthesized by the plant-pathogenic bacterium *Xanthomonas albilineans*. The drug-binding protein AlbA confers albicidin resistance to *Klebsiella oxytoca*. Here we show that AlbA binds albicidin with low nanomolar affinity resulting in full inhibition of its antibacterial activity. We report on the crystal structure of the drug-binding domain of AlbA (AlbAS) in complex with albicidin. Both α-helical repeat domains of AlbAS are required to cooperatively clamp albicidin, which is unusual for drug-binding proteins of the MerR family. Structure-guided NMR binding studies employing synthetic albicidin derivatives give valuable information about ligand promiscuity of AlbAS. Our findings thus expand the general understanding of antibiotic resistance mechanisms and support current drug-design efforts directed at more effective albicidin analogs.

## Abstract (web summary)

The potent antibacterial compound albicidin is synthesized by the plant-pathogenic bacterium *Xanthomonas albilineans*. Here the authors employ a multidisciplinary approach and provide structural and mechanistic insights into how the drug-binding protein AlbA confers albicidin resistance to *Klebsiella oxytoca*.

## Introduction

**Fig. 1:**
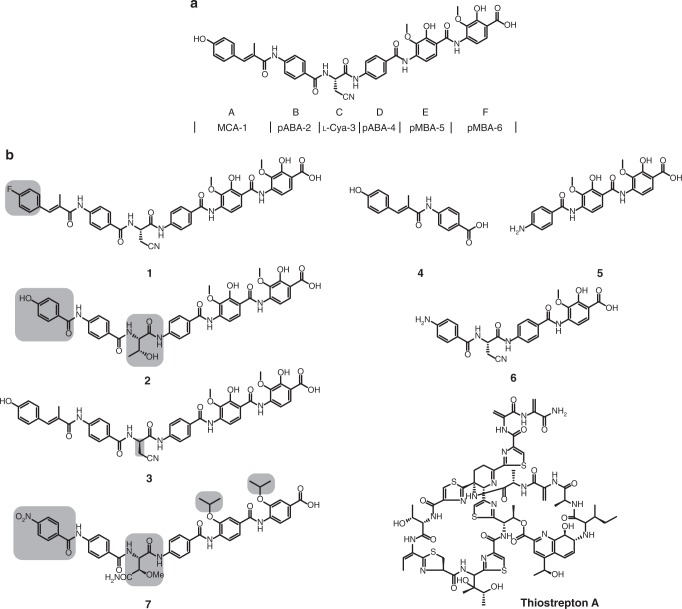
Structure of albicidin. **a** Albicidin is composed of six building blocks (denoted A–F): *p*-coumaric acid (MCA-1); *p*-aminobenzoic acid (*p*ABA-2 and *p*ABA-4); non-proteinogenic α-amino acid l-Cya-3; and two 4-amino-2-hydroxy-3-methoxybenzoic acids (*p*MBA-5 and *p*MBA-6). **b** Overview of tested compounds. Structural variations with respect to albicidin are highlighted in gray

The increasing number of antibiotic-resistant bacteria worldwide has become a major public health threat. Discovery and study of different bacterial resistance strategies as well as the development of new antimicrobial compounds, which are also able to overcome the protecting outer-membrane barrier of Gram-negative bacteria, are of great urgency^1^. Albicidin is a phytotoxic small molecule synthesized by the Gram-negative plant pathogen *Xanthomonas albilineans* that causes leaf scald disease in sugarcane plants^2^. Besides its phytotoxicity, albicidin is bactericidal in the nanomolar range against Gram-positive and in particular against Gram-negative bacteria with low minimal inhibitory concentrations (MIC), e.g., against *Staphylococcus aureus* (4.0 μg mL^−1^), *Salmonella enteritidis* (0.5 μg mL^−1^), *Pseudomonas aeruginosa* DSM 117 (1.0 μg mL^−1^), and *Escherichia coli* (0.063 μg mL^−1^)^3,4^. The molecular target of albicidin is DNA gyrase (topoisomerase II), an enzyme essential for bacterial DNA replication. Albicidin has been reported to block the ATP-dependent DNA cleavage-religation step at the gyrase A subunit^3^. The biosynthesis of albicidin is based on a hybrid polyketide synthase/nonribosomal peptide synthetase^5,6^. The structure of albicidin consists of six building blocks (Fig. 1a): methyl *p*-coumaric acid (MCA-1) at the N terminus; two *p*-aminobenzoic acids (*p*ABA-2 and *p*ABA-4); the unusual non-proteinogenic α-amino acid β-cyano-l-alanine (l-Cya-3) centered in the structure; and two 4-amino-2-hydroxy-3-methoxybenzoic acids (*p*MBA-5 and *p*MBA-6) at the C-terminal end. The structure elucidation^5,^^7^ of albicidin and a total synthesis route^4^ enabled initial structure-activity relationship (SAR) studies^8–10^.

Previously, a number of resistance mechanisms against albicidin have been described: examples include the nucleoside transporter Tsx, for which mutations have been reported that compromise its abilities to import albicidin^11,12^, or the endopeptidase AlbD from *Pantoea dispersa*^13^, which cleaves albicidin into two inactive fragments^14^. Another strategy that counteracts the antibacterial effect of albicidin is drug binding, as it is also known for the tetracycline-binding protein (TetR family)^15^ or the thiostrepton-binding protein (MerR family)^16^. Examples of such albicidin-binding proteins are AlbA from *Klebsiella oxytoca*^17^ and AlbB from *Alcaligenes denitrificans*^18^, which exert protective effects for survival of the host strains. Previous studies on AlbA/AlbB have demonstrated non-covalent binding of albicidin^17,18^. Furthermore, far-ultraviolet (UV) circular dichroism (CD) spectroscopy has indicated a mostly α-helical structure for AlbA^19^. Specific amino-acid residues such as His125 have been suggested to play a role in albicidin binding^19,20^. Due to high similarities to the DNA-binding domains of transcriptional regulator proteins NifA and NtrC (nitrogen regulatory protein) a classification of AlbA/AlbB as transcription activator proteins has been suggested^19^.

Transcriptional regulator proteins are widespread in nature. For instance, the MerR (Mercury Resistance) transcriptional regulator family is known to activate several multi-drug resistance (MDR) systems in response to environmental stress^21^. Members of this family are characterized by a highly conserved N-terminal winged helix-turn-helix (HTH) DNA-binding motif. The HTH motif is connected by a long coiled-coil linker to a C-terminal effector-binding domain that differs in structure and function among the family members^21^. Activation of these regulators occurs in response to oxidative stress and heavy metals or small molecules, e.g., antibiotics, binding to the effector domain^22^. Well-known members of the MerR family are the transcriptional regulators BmrR from *Bacillus subtilis*^22,23^, and SoxR from *Escherichia coli*^24^. Another prominent and well-described MerR system is the thiopeptide-binding protein TipA (thiostrepton-induced protein A) from *Streptomyces lividans*^25,26^. The *tipA* gene encodes for two alternate in-frame translation products: the long form TipAL consisting of the N-terminal HTH DNA-binding domain (TipAN), and the shorter C-terminal thiopeptide drug-binding domain (TipAS)^27^. Drug binding to TipAL leads to an upregulation of its own expression and confers resistance against several thiopeptide antibiotics, e.g., thiostrepton, nosiheptide, and promothiocin A^16^. Previously, Grzesiek and co-workers have solved the solution structure of TipAS bound to various thiopeptides^16,27^, which for the first time gave insights into binding determinants and domain dynamics of the protein.

In this work, we characterized the MerR-like drug-binding protein AlbA that displays a high binding affinity to albicidin. We elucidated the crystal structure of the complex between albicidin and the drug-binding domain AlbAS, demonstrating that this domain is structurally distinct from other drug-binding domains of MerR family members, such as BmrR^23^ or TipAS^27^. The unusual topology of AlbAS comprises two tandem domains that are required to wrap around and fully enclose the drug in the core of the protein. Nuclear magnetic resonance (NMR), CD, and fluorescence spectroscopic measurements provided detailed insights into the binding mechanism and the structural elements required for drug binding of AlbA. Consistent with our structural data, binding studies on synthetic albicidin derivatives revealed ligand promiscuity of AlbAS for aromatic acylpentapeptides as well as further supported our model of the binding mechanism. Our findings represent valuable knowledge about antibiotic capture as an important resistance mechanism and how to possibly circumvent it in the case of albicidin.

## Results

### AlbA is a MerR-like transcriptional regulator

A sequence similarity search using Basic Local Alignment Search Tool (BLAST) for AlbA (accession number (AC): Q8KRS7)^17^ and AlbB (AC: Q44567)^18^ suggests that they belong to the MerR family of transcriptional regulators (*E*-values of 10^−160^ (AlbA) and 10^−37^ (AlbB)). Moreover, AlbA shows high structural homology to the thiopeptide-binding protein TipAS (Supplementary Figure 1). Further homologous proteins were identified in various Gram-negative bacteria belonging to the family of Enterobacteriaceae like *Raoultella ornithinolytica*, *Leclercia adecarboxylata*, or *Enterobacter mori* (Supplementary Figure 2A and Supplementary Figure 3). Bioinformatic comparison of AlbA and AlbB showed that while being distantly related in primary structure, both proteins most probably share high secondary structure similarity (Supplementary Figure 2B). A striking difference between both proteins is the isoelectric point (AlbA: pI = 4.99, AlbB: pI = 10.15) due to the reversed content of acidic (15% for AlbA versus 8% for AlbB) and basic amino-acid residues (10% for AlbA versus 15% for AlbB).

Bioinformatic analysis of the *K. oxytoca* and *A. denitrificans* genomes revealed a putative HTH motif in AlbA and AlbB that might be involved in DNA binding. This domain is conserved in several MerR-transcription activators^21^. In analogy to the TipA system^27^, we assumed that the *albA*/*albB* genes each encode for two proteins: a full-length protein and a truncated protein as a result of two in-frame translation products. Based on the TipA nomenclature^27^, we termed the full-length versions AlbAL/AlbBL (AlbAL 40 kDa, 348 amino acids, AC: WP_016808841). Hence, AlbAL/AlbBL consist of the MerR-characteristic N-terminal HTH DNA-binding domain and the C-terminal drug-binding domain AlbAS/AlbBS. Sequence alignments with TipAL and secondary structure prediction illustrated that AlbAS (25.8 kDa, 221 amino acids) may comprise an unusual tandem arrangement of two TipAS-like drug-binding domains (Supplementary Figure 1). Interested in the capture mechanism toward albicidin, we subsequently focused on AlbAS and validated the results by employing AlbAL in key experiments (Supplementary Figure 4A).

### AlbA captures albicidin with low nanomolar binding affinity

**Fig. 2:**
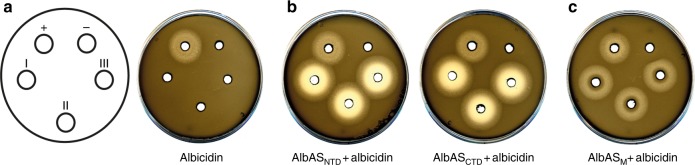
Agar diffusion assays. **a**–**c** AlbAS, AlbAS_NTD_, AlbAS_CTD_, and AlbAS_M_ (40 µM) with 40 μM albicidin (in triplicates I–III). The first plate illustrates the sample arrangement on the assay plates. Positive control with only 40 µM albicidin (+) and negative control with 40 µM protein in 5% DMSO and buffer (−) are shown on top. Representative results are shown for three independent experiments

Initial in vitro agar diffusion assays with *E. coli* and a pre-incubated AlbA-albicidin mixture clearly demonstrated the albicidin-neutralizing effect of both AlbAS (Fig. 2a) and AlbAL (Supplementary Figure 5A).

MIC values for albicidin were determined against four *K. oxytoca* and seven *Klebsiella pneumoniae* strains as well as several ESKAPE organisms such as *P. aeruginosa*, *R. ornithinolytica*, *Acinetobacter baumanii*, and *Enterobacter cloacae*. All strains were resistant against albicidin with MIC values of ≥64 µg mL^−1^. By bioinformatic analysis, we identified AlbA homologs in many of these pathogens (Supplementary Figure 3) indicating that AlbA may function as a widespread resistance mechanism.

**Fig. 3:**
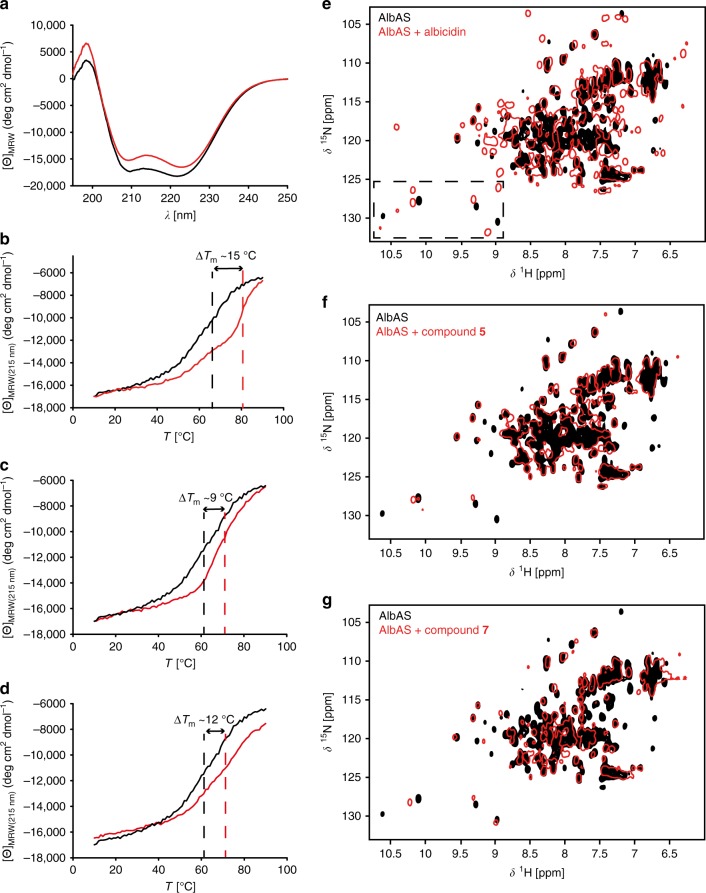
Impact of albicidin binding on structure and stability of AlbAS. **a** Far-UV CD spectra of AlbAS in the absence (black) or presence (red) of albicidin (molar ratio 1.5:1 albicidin:AlbAS) at 21 °C. **b**–**d** CD-detected thermal unfolding of AlbAS in the absence (black) or presence (red) of albicidin, compound **5**, or compound **7**. *T*_m_ values are indicated by dashed lines. For the overlay, the [Θ]_MRW(215 nm)_ signals of AlbAS–albicidin and AlbAS–compound **5** were scaled to that of AlbAS alone using a factor of 1.157 and 1.091, respectively. **e**–**g** Overlay of ^1^H-^15^N HSQC NMR spectra of AlbAS in the absence (black) or presence (red) of albicidin, compound **5**, or compound **7**, respectively. CD experiments were performed once. NMR experiments with the AlbAS–albicidin complex were performed five times and a representative spectrum is shown in **e**. NMR experiments using albicidin derivatives were conducted once

Far-UV CD spectroscopy of AlbAS and AlbAL in the absence and presence of albicidin showed signal minima at wavelengths of 209 and 222 nm, which are characteristic of α-helical secondary structures (Fig. 3a and Supplementary Figure 5B). No significant changes in secondary structure were induced by albicidin binding to both proteins. However, assessing the thermodynamic protein stabilities showed that the melting temperature, *T*_m_, of AlbAS increased significantly upon albicidin binding from 66 to 81 °C (Fig. 3b). Binding of albicidin to AlbAL caused a less-pronounced increase in *T*_m_ from 71 to 74 °C (Supplementary Figure 5C).

We further inspected the structural rearrangement of AlbAS upon albicidin binding via two-dimensional (2D) NMR experiments with ^15^N-labeled AlbAS. The ^1^H-^15^N heteronuclear single-quantum coherence (HSQC) spectrum of AlbAS was characterized by considerable line-broadening and signal overlap (Fig. 3e). Peak picking yielded only 95 of the expected 209 cross-peaks. By contrast, addition of albicidin not only caused chemical shift changes but also the appearance of new cross-peaks (approximately 175 cross-peaks in total). For example, the well-resolved indole region of tryptophans showed three versus nine cross-peaks in the absence and presence of the ligand, respectively. A tentative assignment of these side-chain signals using Trp-to-Phe mutagenesis revealed that at least W27, W56, W133, and W195 became detectable upon albicidin binding (Supplementary Figure 6). Interestingly, the observation of doubled resonances for the side chains of W56 and W133 indicated that these residues adopt two different and slowly interconverting conformations in the albicidin–AlbAS complex (Supplementary Figure 6). The NMR data suggest that the extensive line-broadening for the unbound AlbAS is caused by internal dynamics on a microsecond to millisecond timescale, which are frozen in a more rigid albicidin-bound state.

Size-exclusion chromatography with multi-angle light scattering (SEC-MALS) measurements yielded molecular masses, *M*, of 27.1 and 27.2 kDa for AlbAS in the absence and presence of albicidin, respectively (Supplementary Figure 7A). In contrast to monomeric AlbAS, the full-length protein AlbAL formed a dimer in solution in both the unbound state (*M* of 82.2 kDa) and albicidin-bound state (*M* of 85.5 kDa) (Supplementary Figure 7B). Dimerization has been shown for other MerR proteins as well, enabling DNA promoter binding^28,21^. In agreement with a dimeric state, ^2^H,^15^N-labeled AlbAL yielded rather broad resonance lines in ^1^H-^15^N transverse relaxation-optimized spectroscopy (TROSY) experiments. More importantly, albicidin binding to AlbAL caused many of the characteristic chemical shift perturbations observed for AlbAS–albicidin indicating a very similar binding mode (Supplementary Figure 5D and E).

**Fig. 4:**
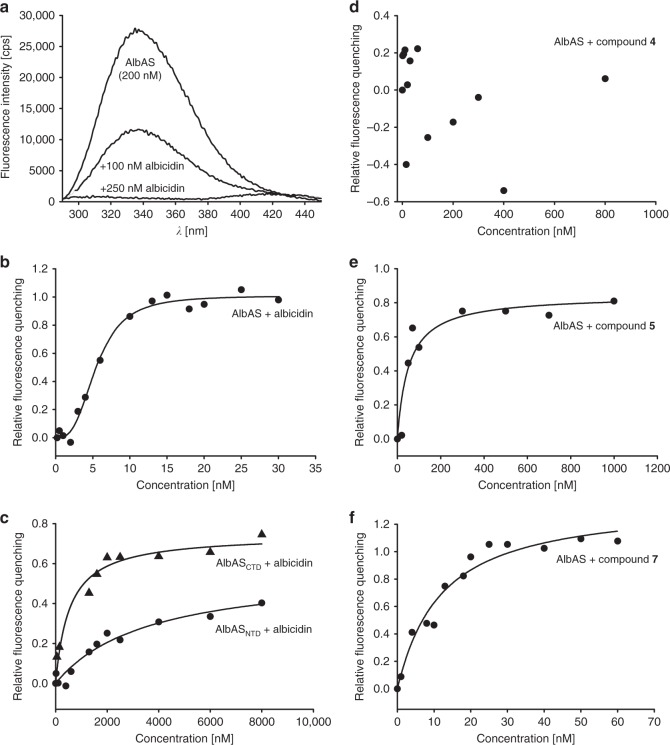
Determination of binding affinities by monitoring quenching of fluorescence emission of AlbAS. **a** Fluorescence quenching of AlbAS (200 nM; *λ*_exc_ = 280 nm) with increasing albicidin concentrations. **b** Nonlinear regression (Eq. (2), see Methods section) of fluorescence quenching data yielding a *K*_d_ of 5.6 ± 0.2 nM and a Hill coefficient, *n*, of 3.0 ± 0.5 for the interaction of albicidin with AlbAS (20 nM). **c** Nonlinear regression of the quenching data yielded *K*_d_ values of 3.4 ± 0.2 and 0.6 ± 0.1 µM for the interaction of albicidin with the dissected domains AlbAS_NTD_ (100 nM) and AlbAS_CTD_ (100 nM), respectively. **d** AlbAS (20 nM) titrated with compound **4** did not lead to significant quenching of the fluorescence emission signal. **e** Titration of AlbAS (20 nM) with compound **5** yielded a *K*_d_ of 55.5 ± 3.6 nM (Eq. (2), see Methods section). **f** Titration of AlbAS (20 nM) with compound **7** yielded a *K*_d_ of 14.0 ± 2.9 nM (Eq. (3), see Methods section). Representative binding curves are shown for two independent experiments each. Errors are given as s.d

In order to estimate the binding strength between AlbAS and albicidin, we monitored characteristic signals in a well-resolved reporter region of the ^1^H-^15^N HSQC spectrum during a titration series (Supplementary Figure 8A). The simultaneous observation of signals arising from both AlbAS and its albicidin-bound state, as well as and the mutual change in populations of these two states indicated a slow-exchange regime in agreement with strong ligand binding in the nM–pM range (Supplementary Figure 8B and Supplementary Figure 8C)^29^. Owing to this very strong interaction and the high protein concentrations required in the NMR experiments, we employed fluorescence spectroscopy to avoid ligand depletion effects and to ultimately determine the dissociation constant, *K*_d_, of albicidin binding to AlbAS and AlbAL. The experimental read-out was based on quenching of fluorescence emission of AlbAS/AlbAL upon binding of albicidin (Fig. 4a). Analysis of the observed binding response for AlbAS yielded a *K*_d_ of 5.6 ± 0.2 nM and indicated positive cooperativity with a Hill coefficient, *n*, of 3.0 ± 0.5 (Fig. 4b). The full-length protein AlbAL showed a similar binding affinity (*K*_d_ of 7.4 ± 0.9 nM) and cooperativity (*n* of 2.0 ± 0.2) (Supplementary Figure 5F). These results demonstrated that the drug-binding domain of AlbA acts as a high-affinity capture system for albicidin.

It has been shown previously that albicidin exhibits a half maximal inhibitory concentration (IC_50_) of 40 nM for its molecular target DNA gyrase^3^. To investigate whether AlbAS is capable of protecting DNA gyrase against albicidin, we performed an NMR-based in vitro competition experiment employing the gyrase construct GyrBA59 from *E. coli* (Supplementary Figure 9A). Purified and enzymatically active GyrBA59 (Supplementary Figure 9B and C) was incubated with MgCl_2_, ATP, supercoiled pBR322 plasmid DNA, and equimolar amounts of AlbAS. An equimolar amount of albicidin was then added to the mixture to allow for direct competition. The overlay of ^1^H-^15^N correlation spectra showed that AlbAS fully populated the albicidin-bound state with no free AlbAS being detectable (Supplementary Figure 9D and E). This demonstrated that AlbAS can efficiently sequester albicidin before the inhibitor targets DNA gyrase.

### The structure of the AlbAS–albicidin complex

**Fig. 5:**
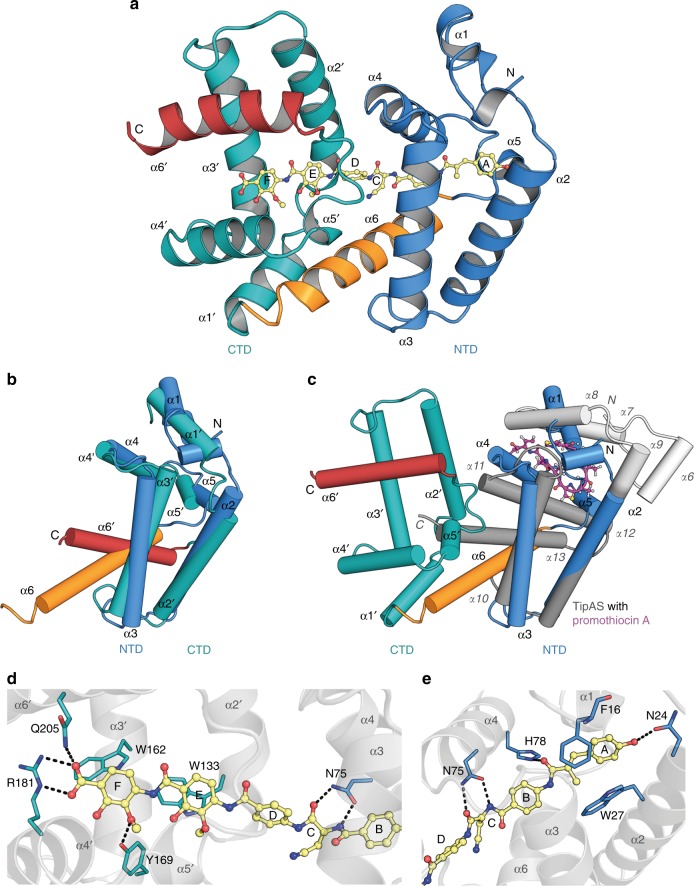
Structure of the AlbAS–albicidin complex and key residues of AlbAS for albicidin binding. **a** AlbAS is shown in cartoon representation with the NTD (α1-α6) in blue and the CTD (α1′-α6′) in cyan. The elongated helix α6 connecting both domains is highlighted in orange. Albicidin is shown in ball-and-stick representation (yellow) and pervades both domains of AlbAS. **b** Superposition of the NTD and CTD of AlbAS. α-helical elements are labeled (a prime indicates assignment to the CTD). The protruding helix α1 of the NTD is predicted to be part of a longer α-helical linker region of AlbAL, which connects the HTH motif and the drug-binding region of the protein. **c** Superposition of the AlbAS–albicidin complex (ligand not shown) and TipAS (PDB 2MBZ)^27^ bound to promothiocin A (ball-and-stick representation, carbon magenta). A region that is unfolded in isolated TipAS and forms an α-helical lid (α6-α9) after drug binding is colored in white. Helices present in the unbound form of TipAS are drawn in dark gray. **d** Interactions of the AlbAS CTD with albicidin portion D-E-F. **e** Interactions of the AlbAS NTD with albicidin portion A-B-C. Residues of the NTD, the CTD, and albicidin are colored in blue, cyan, and yellow, respectively, with oxygens in red and nitrogens in blue. Hydrogen bonds and ionic interactions are indicated by dashed lines

We obtained well-diffracting crystals for the AlbAS–albicidin complex and solved its structure by single-wavelength anomalous dispersion with selenomethionine-labeled AlbAS (Fig. 5a). Model refinement (1.7 Å resolution) converged with excellent statistics and geometry (Supplementary Table 1 and Supplementary Figure 10A and B). The structure of AlbAS could be fully modeled except for the C-terminal residues 215–221. An asymmetric unit contains two copies of the protein that are virtually identical with a root mean square deviation (rmsd) of 0.6 Å for all Cα atoms. AlbAS adopts an all-α-helical fold in agreement with CD spectroscopy (Fig. 3a). As postulated from our bioinformatic analysis (Supplementary Figure 11A and Supplementary Figure 1), the structure of AlbAS is organized in two repeat units, i.e., the N-terminal domain (NTD, residues M1-R112) and the C-terminal domain (CTD, residues Y113-E221). A 24-residue α-helix (P92-R112) connects the NTD with the CTD. To reflect the tandem arrangement in AlbAS, we designated helices of the NTD as α1-α6 and the equivalent helices of the CTD as α1′-α6′ (Supplementary Figure 11A). The NTD and CTD are arranged relative to each other via 2_1_ screw pseudosymmetry and can be superimposed (using boundaries D20-L114 and D128-A215) with an rmsd of 1.7 Å (Fig. 5b and Supplementary Figure 11A).

A DALI^30^ search for structurally related folds resulted in very low *Z*-scores. The most related structure with a *Z*-score of 5.8 is the drug-binding domain TipAS (PDB 2MBZ, in complex with promothiocin A)^27^ with a sequence identity of only 13%. TipAS (G111-P253) superimposes with both the NTD (M1-R112) and CTD (Y113-Q214) of AlbAS with Cα rmsd values of 2.2 and 5.0 Å, respectively. The deviations mainly arise from structural differences at the N-terminal regions, whereas helices α2-α6 of the NTD (D20-L114) and helices α2′-α6′ of the CTD (Y128-A215) can be well aligned to helices α9-α13 of TipAS (Y155-P253) yielding rmsd values of 2.5 and 2.8 Å, respectively.

To test the albicidin-binding potential of the individual domains, we prepared constructs comprising either the NTD (AlbAS_NTD_) or the CTD (AlbAS_CTD_) (Supplementary Figure 4B). Both constructs adopted folded states in solution as judged by ^1^H-^15^N HSQC spectra (Supplementary Figure 12A and B). However, in agar diffusion assays they failed to suppress the antibacterial effects of albicidin (Fig. 2b). This observation can be ascribed to diminished affinities toward albicidin, as fluorescence spectroscopy yielded *K*_d_ values of 3.4 ± 0.2 μM (AlbAS_NTD_) and 0.6 ± 0.1 μM (AlbAS_CTD_), respectively (Fig. 4c).

In contrast to TipAS, which binds its macrocyclic ligands in an N-terminal pocket (Fig. 5c)^27^, the elongated and rather rigid albicidin molecule is accommodated in a central tunnel of AlbAS thereby pervading both, its NTD and CTD. Interestingly, both repeat units utilize an equivalent pocket to harbor the different segments of albicidin, albeit with opposite entrance sites (Supplementary Figure 11B). The binding tunnel is decorated by residues of all helices, except for the N-terminal helix α1 (Fig. 5a), and exhibits a predominantly hydrophobic inner surface. The building blocks A, B, and C of albicidin are surrounded by the NTD of AlbAS, whereas building blocks D, E, and F are enclosed by the CTD. The high-salt crystallization conditions reflect the hydrophobic character of the interaction, which is mediated by several hydrophobic residues pointing into the tunnel, e.g., F16, W27, W56, L60, L71, T88, I95, L130, W133, P134, W162, and W195 (an overview of all interacting residues is given in Supplementary Figure 13). Five out of eight Trp side chains (W27, W56, W133, W162, and W195) line the inner surface of the tunnel. Residues F16, W27, W133, and W162 are *π*-stacked to the aromatic building blocks A, E, and F of albicidin. Besides strong hydrophobic interactions, a salt bridge between the carboxylate moiety of albicidin and R181 of the CTD might play an important role for the orientation and fixation of the ligand in the tunnel (Fig. 5d). Furthermore, several hydrogen bonds contribute to the binding of albicidin. For example, pMBA-6 (building block F) establishes two hydrogen bonds to Y169 and Q205 of the CTD (Fig. 5d), while N24 and H78 of the NTD hydrogen-bond to the hydroxyl and carbonyl moiety of MCA-1 (building block A), respectively (Fig. 5e). Owing to its rigid scaffold with a single α-amino acid, albicidin adopts a V-shaped conformation with the Cα atom of l-Cya-3 at the center and the building blocks A-B-C and D-E-F forming the two arms that span an angle of about 130°. Importantly, the side chain of N75 of AlbAS forms two hydrogen bonds with the amide proton and the carbonyl oxygen of the central l-Cya-3 (*Φ* and *Ψ* values of 66.0° and −128.7°), thereby stabilizing this specific conformer of albicidin. The cyanomethylene side chain of l-Cya-3 is oriented outwards the binding tunnel approaching helices α6 and α1′. Interestingly, the amide proton of *p*ABA-4 is pointing at the triple bond of the nitrile moiety with a distance of about 1.4 Å.

Besides the mainly hydrophobic character of the interaction between AlbAS and albicidin, we propose that N75, R181, and Q205 are important residues for recognition of albicidin. Based on the crystal structure we generated the triple variant AlbAS_M_ with amino-acid substitutions N75A, R181A, and Q205A (Supplementary Figure 4A). Employing the agar diffusion assay as a first line of evidence, AlbAS_M_ was indeed not capable of efficiently suppressing the antibacterial activity of albicidin (Fig. 2c), albeit binding was not fully impeded (Supplementary Figure 14).

### AlbAS tolerates structural variations of albicidin

The above-described interaction between AlbAS and albicidin raised further questions on the tolerance of AlbAS toward structural variations of albicidin and thus the possible design of albicidin analogs, which could escape arrest by AlbAS. Based on previous SAR studies, we were equipped with a number of synthetic albicidin derivatives and substructures thereof^8,9^. We employed the agar diffusion assay with *E. coli* and ^1^H-^15^N HSQC experiments in order to monitor the ability of AlbAS to bind albicidin analogs (Supplementary Figure 14 and 15).

From our library of albicidin-derived compounds we chose seven derivatives (compounds **1**–**7**) as well as the TipAS-specific ligand thiostrepton A as a control substance (Fig. 1b). These albicidin derivatives comprised variations of building blocks A and C, which otherwise stabilize the AlbAS–albicidin complex through hydrogen bonds to N24 and H78 (building block A), N75 (l-Cya-3, building block C), as well as *π*–*π* stacking and hydrophobic interactions (building block A) to W27 of AlbAS. In compound **1**, a fluorine atom replaced the phenolic hydroxyl group of the coumaric acid (building block A), thus eliminating hydrogen bond capabilities to N24. The agar diffusion assay showed that the presence of AlbAS abolished the antibacterial activity of compound **1** (Supplementary Figure 15). Moreover, the HSQC signal pattern clearly indicated strong binding by AlbAS similar to that of albicidin (Supplementary Figure 16).

Compound **2** bore two structural modifications: (i) it was shorter than albicidin due to a substitution of coumaric acid by a para-hydroxy benzoic acid moiety; and (ii) l-Cya-3 was substituted by the sterically more demanding l-Thr with H-bond donor and acceptor capabilities. Despite the expectedly missing hydrogen bond to residue N24 and the variation in the side chain of l-Cya-3 (building block C), the agar diffusion assay indeed confirmed binding of the strongly antibacterial compound **2** (Supplementary Figure 15). Accordingly, we observed the characteristic HSQC signal pattern as in the case of albicidin binding to AlbAS (Supplementary Figure 14 and 16). Apparently, both structural modifications (building blocks A and C) had no significant influence on binding by AlbAS.

In order to assess the importance of the only stereocenter in albicidin for binding to AlbAS, we also tested the antibacterially active enantiomer of albicidin^4^ bearing d-Cya-3 (compound **3**). Remarkably, AlbAS was still able to bind compound **3** (Supplementary Figure 14) and thus to prohibit its antibacterial effects (Supplementary Figure 15).

Finally, we tested truncated albicidin derivatives (compounds **4**, **5**, and **6**) to evaluate binding contributions from the NTD and CTD (Fig. 1b). Compound **4** only consisted of the building blocks A and B, and therefore addressed binding contributions from the NTD of AlbAS. On the contrary, compound **5** comprised only the C-terminal building blocks D, E, and F, and was thought to potentially accommodate in the CTD of AlbAS. Ultimately, compound **6** was devoid of building blocks A and F and was thus hypothesized to miss essential interactions with residues N24, W27, W162, Y169, R181, and Q205 in the NTD and CTD. The truncated albicidin variants **4**, **5**, and **6** did not display antibacterial activity (Supplementary Figure 15), however, the change in the NMR signal pattern clearly demonstrated interaction between AlbAS and the respective derivatives (Supplementary Figure 14). Notably, while compounds **5** and **6** again caused spectra in the slow-exchange regime (strong binding), compound **4** showed a gradual chemical shift change typical for fast-exchange systems (weak binding, Fig. 4d). Fluorescence quenching experiments with AlbAS and compound **5** resulted in a *K*_d_ of 55.5 ± 3.6 nM (Fig. 4e). Compound **4** had no consistent effect on fluorescence emission spectra of AlbAS suggesting a very low binding affinity (Fig. 4d). Taken together, these results underline the importance of the CTD as a fixation point for albicidin.

Facing the high structural similarities between albicidin and the anti-Gram-negative compounds cystobactamid^31^ and coralmycin^32^, we tested binding capabilities of an albicidin derivative (compound **7**)^33^ with cystobactamid-like features (Fig. 1b). The pattern of NMR reporter signals of AlbAS clearly pointed to a strong binding of compound **7** in the nanomolar *K*_d_ range. Our fluorescence quenching experiments revealed a *K*_d_ of 14.0 ± 2.9 nM for compound **7** (Fig. 4f), which is similar to the binding affinity of AlbAS toward albicidin (5.6 ± 0.2 nM), however, no cooperative behavior was observed. Analysis of the agar diffusion assay showed that AlbAS is able to neutralize the antibacterial effects of compound **7**, albeit with decreased efficiency (Supplementary Figure 15). Finally, thiostrepton A was devoid of any binding affinity toward AlbAS (Supplementary Figure 15 and Supplementary Figure 14).

## Discussion

AlbA has been reported as a binding protein conferring resistance against the potent antibiotic albicidin^17,19,20^. Previous studies already mentioned a single high-affinity binding site for albicidin in AlbAS^20,34^, and an α-helical structure^19^, but they lacked both the knowledge of the chemical structure of albicidin and the high-resolution structure of AlbAS. Our antibacterial tests using an in vitro agar diffusion assay combined with biophysical binding studies via CD, NMR, and fluorescence spectroscopy underscore the high binding affinity of AlbAS and AlbAL toward albicidin (*K*_d_ of 5.6 ± 0.2 and 7.4 ± 0.9 nM, respectively) and other derivatives. The sensitivity of fluorescence quenching experiments was essential for the determination of such a low *K*_d_ value. However, as we were restricted to minimum protein concentrations of 20 nM, the determined *K*_d_ values could still represent upper boundaries. Therefore, the previously reported *K*_d_ of 64 nM^34^ is at least one order of magnitude to high. Surprisingly, quenching of AlbAS emission by albicidin proved to be highly efficient (100% quenching) possibly reflecting the network of tryptophans in the binding tunnel, in particular in that of the CTD (Fig. 5c).

Competition experiments with the DNA gyrase construct GyrBA59 and albicidin (Supplementary Figure 9D and E) demonstrated the strong binding affinity of AlbAS and its ability to protect the molecular target from inhibition. In light of previously determined IC_50_ values of 40 nM for DNA gyrase, we suggest that AlbA may either be more abundant in the cell than DNA gyrase and/or exhibit superior association rates *k*_on_ to efficiently capture albicidin after cell entry.

The crystal structure of the drug-binding domain AlbAS revealed a tandem architecture with the NTD and CTD representing structural repeats of each other that are connected by the extended helix α6 of the NTD (Fig. 5a). Intriguingly, nature has adapted to the elongated and fairly rigid scaffold of albicidin by combining two TipAS-type folding units to fully enclose the highly potent toxin. Albicidin is thus clamped between the NTD and CTD and stabilized predominantly by hydrophobic and *π*–*π* stacking interactions (Fig. 5d, e, Supplementary Figure 11A and B). Based on our structural data, we can exclude the previously suggested direct involvement of residue H125 in albicidin binding^20^, since this residue is located in helix α1′ and oriented toward the protein exterior (Supplementary Figure 17). Several hydrogen bonds, and in particular the salt bridge between the carboxylic acid of albicidin and R181 of AlbAS, most likely confer ligand selectivity. We assume that R181 is relevant to orient albicidin in the binding cleft by anchoring the carboxylic acid of *p*MBA-6 (building block F) in the CTD.

Attempts to crystallize AlbAS in the absence of albicidin did not succeed. According to this, the increase in observable signals in ^1^H-^15^N HSQC spectra of AlbAS upon albicidin binding suggests that free AlbAS displays inherent protein dynamics on the micro-to-millisecond timescale. Based on the repeat structure of AlbAS with few direct contacts between the repeat units even in the albicidin-bound state, we hypothesize that albicidin may fix the otherwise flexible relative orientation of the NTD and CTD. In addition, dynamics within the binding tunnel in the absence of albicidin may also account for some of these observations, as only three out of eight expected HSQC signals for Trp side chains were detected with good signal-to-noise ratio (Supplementary Figure 6 and 16). Ligand-induced stabilization has also been observed for the structurally related drug-binding protein TipAS^16^, for which binding of thiopeptides induces an otherwise non-observable α-helical lid to pack onto the ligand (Fig. 5c)^27^. In contrast, however, the comparison of AlbAS/AlbAL and the AlbAS/AlbAL–albicidin complexes by CD spectroscopy showed that the α-helical content was virtually independent of ligand binding (Fig. 3a and Supplementary Figure 5B). The dynamic nature of the vacant protein suggests a possible binding mechanism: AlbAS exploits its inherent structural plasticity in order to clamp the elongated and rather rigid albicidin molecule in a buried tunnel of about 33 Å in length that is built from both repeat units (Supplementary Figure 18). The observed positive cooperativity with Hill coefficients *n* of 2.0–3.0 is in agreement with a binding process that includes recruitment of albicidin to one domain favoring subsequent association to the neighboring domain. Such a cooperative binding behavior has been also observed for the MerR protein BmrR^35^. The strong binding of albicidin consequently leads to a well-defined NTD-CTD arrangement in AlbAS causing an increase in thermal stability, Δ*T*_m_, of about 15 °C.

Our concept of ligand-mediated domain-domain bridging is supported by binding effects caused by albicidin fragments: despite strong binding to AlbAS (*K*_d_ of 55.5 ± 3.6 nM), the C-terminal segment of albicidin (compound **5**) did not cause the appearance of additional resonances in the HSQC spectrum of AlbAS, and neither did compounds **4** and **6** (Supplementary Figure 14). This observation demonstrates that the fragments were not capable of efficiently stabilizing the NTD-CTD arrangement (Supplementary Figure 18), while all tested full-length analogs of albicidin did (Supplementary Figure 14). Intriguingly, the full-length cystobactamid-type analog (compound **7**) was not able to stabilize AlbAS to a similar extent as albicidin (Fig. 3d, g). Despite binding with low nanomolar affinity (*K*_d_ of 14.0 ± 2.9 nM) similar to albicidin, compound **7** showed no positive cooperativity (Fig. 4f). Moreover, compound **7** provoked chemical shift patterns in HSQC spectra of AlbAS reminiscent of those of the C-terminal albicidin fragment (compound **5**). We could not achieve crystallization of the AlbAS–compound **7** complex either. Taken together, these observations clearly support that cystobactamid is mainly bound to the CTD of AlbAS without properly bridging the NTD and CTD. As AlbAS can tolerate a shortened building block A (para-hydroxy benzoic acid in compound **2**, see Supplementary Figure 14), the steric demand of β-methoxy-l-Asn and its anticipated effect on the backbone conformation in building block C appears to hinder the stabilization of both domains. In a previous study, we identified various naturally occurring derivatives of albicidin, amongst them a less active variant with a β-methoxy-l-Asn residue^33^. Hence, this modification may represent a natural mechanism against AlbA-mediated transcriptional regulation.

Truncated AlbAS variants composed of a single domain were not sufficient to properly arrest albicidin: AlbAS_NTD_ and AlbAS_CTD_ were not able to neutralize albicidin in the agar diffusion assay (Fig. 2b) due to their much weaker binding affinities. Fluorescence quenching experiments yielded *K*_d_ values for albicidin binding of 3.4 ± 0.2 µM (NTD) and 0.6 ± 0.1 µM (CTD), respectively, indicating that recruitment of albicidin to AlbAS may be guided by the CTD. We propose that a binding pathway from the CTD toward the NTD of AlbAS would ensure that albicidin is first fully trapped in AlbAS before the binding signal is relayed to the N-terminal HTH domain of full-length AlbAL to induce DNA binding. The high affinity of AlbAS toward the C-terminal fragment of albicidin (compound **5**) further supports the idea of the CTD as an initial anchoring point (Supplementary Figure 18).

Interestingly, when comparing AlbAS with AlbBS only key residues W162, Y169, and R181 of AlbAS are conserved in AlbBS (Supplementary Figure 2B). These residues interact with *p*MBA-6 (building block F) of albicidin indicating that AlbBS might recognize this C-terminal *p*MBA-motif, but that its cognate ligand might otherwise deviate from the albicidin structure. In light of the reduced affinity of AlbAS toward the cystobactamid-type compound **7**, the missing binding cooperativity and the reduced stabilization effect we propose that AlbAS has been evolutionarily optimized to capture albicidin rather than the related cystobactamids or coralmycins^31,32^. Whether AlbBS represents a corresponding cystobactamid- or coralmycin-binding protein will be the subject of future investigations.

Sequence alignments and secondary structure predictions demonstrate the widespread occurrence of AlbAS homologs with tandem architecture among several ESKAPE pathogens (Supplementary Figure 3). These homologs are sequentially different (≤58% difference to AlbAL) but are predicted to adopt a similar α-helical repeat structure in which key residues important for albicidin binding are conserved. The AlbA-mediated sequestration of albicidin might thus operate in clinically relevant pathogens, which showed resistance against albicidin (MIC value ≥64 µg mL^−1^). However, at this point it is unclear to which extent albicidin capture by AlbA homologs contributes to the overall antibacterial resistance in vivo, as several resistance strategies will act in concert. Likewise, it will be necessary to identify full-length AlbAL and the truncated AlbAS species in vivo, and to quantify their cellular abundances.

Given the ligand promiscuity of the TipAS system^27,36^, and other MerR effector-binding domains (i.e., BmrR)^37^, we studied whether AlbAS can accept structurally different albicidin derivatives by using an in vitro agar diffusion assay and an HSQC-based binding assay. While the former assay only provided information on the AlbAS-mediated neutralization of antibacterial derivatives, the NMR experiments gave a direct read-out on the binding capabilities of AlbAS. The synthetic albicidin derivatives submitted to these assays probed steric as well as electronic requirements for effective binding by AlbAS.

As discussed above, the albicidin fragments could not efficiently fix the two domains of AlbAS. This observation supports the suggested clamping mechanism, for which albicidin derivatives are required that can span the entire binding tunnel, thus connecting the NTD and CTD (Supplementary Figure 18).

Structural variations at the N terminus of albicidin (building block A), like the withdrawal of hydrogen bond capabilities (compound **1**) or the replacement of methyl coumaric acid with para-hydroxy benzoic acid (compound **2**) had no significant effect on AlbAS binding. In addition, a polar l-Thr instead of l-Cya-3 (compound **2**) was similarly tolerated by AlbAS (Supplementary Figure 16). Remarkably, as shown for the albicidin enantiomer (compound **3**), inversion of the only stereocenter of the molecule was not detrimental to recognition by AlbAS. The agar diffusion assay clearly demonstrated the neutralizing effect of AlbAS toward compound **3** and the characteristic signal patterns in the HSQC spectra confirmed a binding mode equivalent to that of albicidin (Supplementary Figure 16). AlbAS binding of the enantiomeric compound **3** might be explained based on the orientation of albicidin in the crystal structure: the side chain of l-Cya-3 points out of the binding pocket toward the protein surface (Fig. 5a). The surrounding helices α1′, α4, and α6 would leave sufficient space for d-Cya-3 while preserving the overall binding mode of albicidin. These findings show that the rather flexible AlbAS tolerates structural modifications of the oligo-aromatic scaffold of albicidin.

A connection between AlbA and transcriptional regulator proteins has previously been suggested by Weng et al.^19^ and could be corroborated by our bioinformatics analysis (Supplementary Figure 1). The structural homology to TipAS (Fig. 5c) suggests that AlbAS is a member of the MerR-type transcriptional regulator family. The N-terminal HTH DNA-binding domain requires future studies on the mechanism of transcriptional regulation by AlbAL. This includes questions on how signal transduction within AlbAL is triggered by albicidin binding to its drug-binding domain. Our studies on the full-length protein AlbAL (Supplementary Figure 5) already indicated that the binding mode of albicidin in AlbAS is equivalent to that in AlbAL (Supplementary Figure 5A and F). A major difference between the two systems is the dimerization of AlbAL crucial for DNA binding, in contrast to the monomeric state of AlbAS (Supplementary Figure 7). In both cases, quaternary structure is albicidin-independent illustrating that the binding stimulus is relayed from the NTD toward the HTH motif to trigger promoter binding and in consequence the expression of, e.g., MDR transporters^21^.

For the TipAL system it has been suggested that the (partially) unfolded N-terminal region in unbound TipAS becomes folded upon thiopeptide binding. Formation of this α-helical lid (α6-α9) stabilizes the ligand and forwards the signal to the HTH DNA-binding domain^27^. Owing to the different ligand-binding sites and the different structural organizations of the TipAS and AlbAS N termini (Fig. 5c), the mechanisms of intramolecular signal transduction might differ for these two systems and need to be further investigated. Importantly, the proposed binding pathway proceeding from the CTD toward the NTD and finally the HTH motif may ensure a cooperative coupling between the drug-binding stimulus and DNA binding only when the cognate ligand is fully arrested. To avoid AlbA-mediated resistance, two cases therefore need to be differentiated in drug development of albicidin, i.e., analogs that (i) are not prone to AlbAS capture at all or (ii) are still bound by AlbAS but do not trigger transcriptional regulation (Supplementary Figure 18). Future studies are envisaged to answer these questions.

In summary, our findings contribute to a general understanding of resistance strategies of bacteria against antibiotics. The knowledge on AlbA and homologous members of this widespread class of binding proteins will be generally useful for future antibiotic-development efforts. Depending on their pharmacophores, potent antibiotics could be designed such that they either completely bypass capture or that they do not trigger regulative effects on a cellular level despite being partially arrested by AlbA-like drug-binding proteins.

## Methods

### Bioinformatics

The BLAST^38^ was used for a protein sequence homology search and preparation of a phylogenetic tree^39^. Secondary structure predictions were performed with PSIPRED and PROMALS3D^40,41^.

### Cloning

A codon-optimized synthetic *albAS* (Supplementary Table 2) coding region (Thermo Scientific GmbH, Schwerte, Germany) was cloned into an Amp^R^-pMAT vector system, using *Nde*I and *Not*I restriction enzyme sites. The plasmid was amplified in *E. coli* BL21 DH5α, extracted, double digested with *Nde*I and *Not*I, and the released fragment was cloned into expression vector pET-28a (+) for production of AlbAS bearing an N-terminal, TEV-cleavable His_6_-tag. Codon-optimized synthetic *albAL* (Supplementary Table 2, Thermo Scientific GmbH) was cloned by Gibson cloning^42^ into an expression vector pET-28a (+) for production of AlbAL bearing an N-terminal, TEV-cleavable His_6_-tag.

The amino-acid substitutions (N75A, R181A, and Q205A) in the triple variant AlbAS_M_ as well as the substitutions for the Trp-to-Phe variants of AlbAS (W5F, W27F, W56F, W110F, W133F, W162F, W195F, and W203F) were inserted by Gibson assembly cloning^42^. To this end, one primer pair (Supplementary Table 3) was designed to carry the point mutation in the respective base triplet and to amplify one-half of the gene. The other primer pair amplifies the remaining vector and possesses a 16 bp overhang matching to the 3′-end of the other linear construct. Both constructs were ligated with *Taq* DNA ligase (New England Biolabs GmbH, Frankfurt am Main, Germany) after purification with gel electrophoresis by incubation in the Gibson mixture. The mutated plasmid was then transformed into *E. coli* DH5α cells by heat shock. Transformants were selected on Luria Bertani (LB)-agar plates supplemented with 50 μg mL^−1^ kanamycin and confirmed later by sequencing of the gene.

The AlbAS_NTD_ and AlbAS_CTD_ constructs were cloned using the Gibson assembly method^42^ with the primer pairs listed in Supplementary Table 3. The boundaries for the truncated AlbAS variants (AlbAS_NTD_ M1-E132 and AlbAS_CTD_ D128-E221) were based on bioinformatic analysis before solving the AlbAS crystal structure.

### Protein synthesis and purification of AlbAS and AlbAL

All genes including *albAS*, *albAL*, *albAS*_*M*_, *albAS*_*NTD*_, *albAS*_*CTD*_, and the Trp-to-Phe mutants were expressed in *E. coli* BL21-Gold (DE3) strain (Merck Millipore, Darmstadt, Germany) with the T7 promoter expression system pET-28a (+) bearing an N-terminal TEV-cleavable His_6_-tag in LB medium. The expression was induced with 0.2 mM isopropyl β-d-1-thiogalactopyranoside (IPTG, Biomol, Hamburg, Germany) and the cells were further incubated at 18 °C, 180 rpm for 18 h. For isotope labeling, cells were grown in M9-minimal medium containing ^15^N ammonium chloride (1 g L^−1^), ^13^C_6_ d-glucose (3 g L^−1^), and/or deuterium oxide. Cells were harvested by centrifugation and the resuspended cell pellet (50 mM Tris-HCl, 500 mM NaCl, and 5% glycerin, pH 7.5) was lysed by one passage through a high-pressure cell disrupter TS 0.75 KW (Constant Systems Limited, Königswinter, Germany) at 18 000 psi and incubated with DNAse (3 U per mL lysate). Subsequently, cell lysates were centrifuged at 55 000 × *g* for 30 min to separate the cell debris from soluble protein. The crude supernatant was filtered through a 45 µm filter membrane before loading on the purification column. AlbAS/AlbAL purification was performed by affinity chromatography on an ÄKTApurifier system (GE Healthcare, Munich, Germany) by making use of the N-terminal His_6_-tag binding to a Nickel His-Trap Fast Flow Crude 1 mL column (GE Healthcare). The washing buffer consisted of 50 mM Tris-HCl and 300 mM NaCl at pH 7.5. Then AlbAS/AlbAL was eluted with a buffer containing 50 mM Tris-HCl, 300 mM NaCl, and 500 mM imidazole. To remove imidazole the protein fractions were diluted with the washing buffer and concentrated in centrifugal filter units (Merck, Darmstadt, Germany) with a cutoff of 10 000 nominal molecular weight limit. Cleavage of the His-tag was performed for 2 h at room temperature and 16 h at 4 °C with the addition of 100 μM of purified TEV protease. The separation of the protein from the cleaved His-tag was performed by a second purification step employing the Nickel His-Trap column as described before. Desalting and buffer exchange was accomplished by size-exclusion chromatography on a Superdex High Load 16/60 75 column (GE Healthcare) in the final buffer used for NMR studies (sodium hydrogen phosphate 50 mM and 100 mM NaCl, pH 6.8). The collected fractions were analyzed by SDS-polyacrylamide gel electrophoresis (SDS-PAGE) and the protein concentration was determined on a nano-photometer P330 (Implen, Munich, Germany). For long-time storage, the aliquots (0.5 mL) of protein samples were shock-frozen in liquid nitrogen and stored at −80 °C.

Selenomethionine-labeled protein was produced in 500 mL M9-minimal medium with addition of 25 mg selenomethionine, 50 mg lysine, 50 mg threonine, 50 mg phenylalanine, 25 mg leucine, 25 mg isoleucine, and 25 mg valine shortly bevor induction with 0.2 mM IPTG (at OD_600_ of 0.7–0.8). Cells were incubated at 18 °C, 180 rpm for 20 h^43^. Purification was carried out as described above for wild-type AlbAS. The final buffer contained 50 mM Tris (pH 7.5), 100 mM NaCl, and 4 mM dithiothreitol (DTT).

### Preparation of GyrBA59

The *E. coli* fusion protein GyrBA59, combining the full-length GyrB and a C-terminally truncated GyrA segment (59 kDa), was cloned into pET-28a (+) by restriction/ligation (forward and reverse primers are listed in Supplementary Table 3) using the restriction enzyme sites *Nde*I and *Xho*I. GyrBA59 was expressed in *E. coli* BL21 DE3 over 18 h at 18 °C and 250 rpm using auto induction medium. The cells were harvested and resuspended in buffer 1A (20 mM Tris, 150 mM NaCl, 10 mM imidazole, 10% glycerol, 2 mM DTT, EDTA-free protease inhibitor cocktail (cOmplete, Roche), and DNase 2 U per mL lysate, pH 8.0). After cell lysis with 14 000 psi using a high-pressure cell disrupter TS 0.75 KW (Constant Systems Limited, Königswinter, Germany) the lysate was centrifuged at 50 000 × *g* at 4 °C for 30 min and subsequently incubated with DNAse (2 U per mL lysate) for another 30 min at 4 °C. The protein was purified in four steps as follows: (1) cell lysate was loaded onto a His-Trap HP column and washed back to baseline in buffer 1B (20 mM Tris, 500 mM NaCl, 10 mM imidazole, 10% glycerol, 2 mM DTT, and EDTA-free protease inhibitor cocktail, pH 8.0) before being eluted with a gradient from buffer 1B into buffer 1C (20 mM Tris, 300 mM NaCl, 300 mM imidazole, 10% glycerol, 2 mM DTT, and EDTA-free protease inhibitor cocktail, pH 8.0) over 15 column volumes. (2) Pooled fractions were equilibrated against buffer 1A and digested with TEV protease (1 mg per 10 mg of protein) overnight at 4 °C to cleave the N-terminal 6x His-tag. The mixture was loaded onto a His-Trap HP column and the tag cleavage protein was recovered from the flow through in buffer 2A (20 mM Tris, 10 mM NaCl, 5% glycerol, 2 mM DTT, and EDTA-free protease inhibitor cocktail, pH 8.0). (3) The protein fractions were equilibrated against buffer 2A and loaded onto a Heparin Sepharose column to be eluted with a gradient from buffer 2A into buffer 2B (20 mM Tris, 1 M NaCl, 10% glycerol, 2 mM DTT, and EDTA-free protease inhibitor cocktail, pH 8.0) over 15 column volumes. (4) Fractions from step 3 were concentrated using a 100 kDa cutoff filter unit, before being loaded onto a Superdex 200 HiLoad 16/60 column (GE Healthcare) equilibrated and eluted in buffer 3A (20 mM Tris, 100 mM KCl, 1 mM DTT, and 10% glycerol, pH 8.0). The collected fractions were analyzed by SDS-PAGE and the protein concentration was determined on a nano-photometer P330 (Implen, Munich, Germany). Post size-exclusion fractions were pooled, concentrated, and stored at −80 °C.

### DNA cleavage assay

*E. coli* DNA gyrase cleavage assays were performed by incubating 250 ng of supercoiled pBR322 plasmid DNA (Inspiralis, Norwich, UK) with 25 nM of GyrBA59 protein in 20 µL reactions containing 35 mM Tris-HCl (pH 8.0), 24 mM KCl, 4 mM MgCl_2_, 2 mM DTT, 1.8 mM spermidine, 1.4 mM ATP, 6.5% (w/v) glycerol, and 0.1 mg mL^−1^ bovine serum albumin in the presence or absence of various concentrations of albicidin (final concentration of 5% dimethyl sulfoxide (DMSO), incubation at 37 °C for 30 min). Linearized DNA was released by further incubation with 0.2% (w/v) SDS and 0.1 mg mL^−1^ proteinase K (Promega, Madison, USA) at 37 °C for 30 min. The reaction was stopped by addition of 20 µL of reaction stop buffer (STEB buffer) (40% (w/v) sucrose, 100 mM Tris-HCl (pH 8.0), 10 mM EDTA, and 0.5 mg mL^−1^ Bromophenol Blue) and precipitation using 30 µL of chloroform/isoamyl alcohol 24:1 (v:v). Samples were analyzed by electrophoresis in 1% w/v agarose gel containing 0.5 mg mL^−1^ ethidium bromide followed by photography under ultraviolet illumination.

### MIC assay

The MIC assay was performed by Antiinfectives Intelligence GmbH (Rheinbach, Germany) using internationally standardized protocols and employing clinical isolates of *K. oxytoca, K. pneumoniae, R. ornithologica, P. aeruginosa, A. baumannii* and *E. cloacae*.

### Chemical synthesis

Synthesis protocols and analytical data have been recently described for albicidin^9^, compound **1**^8^, compounds **3**, **5**, and **6**^14^ as well as compounds **4** and **7**^33^. The coupling of the N-terminal building block and the global deprotection of compound **2** are described in the Supplementary Methods part 1. Thiostrepton A was purchased from Merck (Darmstadt, Germany).

### Agar diffusion assay

To verify the neutralizing ability of AlbAS, AlbAL, AlbAS_M_, AlbAS_CTD_, or AlbAS_NTD_ against different compounds, agar diffusion assays with the respective compounds were performed. An overnight culture of *E. coli* DH5α was diluted to an OD_600_ of 0.05 with 0.75% LB-agar. The protein-albicidin reaction mixture consisted of a 1:1 mixture of 40 μM albicidin (or derivatives) with 40 μM protein (either AlbAS or its variants AlbAL, AlbAS_M_, AlbAS_NTD_, and AlbAS_CTD_). The mixture was incubated for 20 min at room temperature in the dark. Reaction mixtures of 30 μL were transferred in triplicates of 2 mm holes on the inoculated agar plate. The plates were evaluated concerning growth-inhibition zone after incubation for 18 h at 37 °C.

### CD spectroscopy

For determination of the melting temperature and information on protein-ligand stability, CD measurements were performed on a J-815 CD spectrometer (Jasco, Groß-Umstadt, Germany). Protein samples (2.0 mM for AlbAS and 0.2 mM for AlbAL) were saturated with 1.5-fold albicidin and diluted to a protein concentration of 20 μM in order to ensure a final DMSO content of only 0.05% for AlbAS and 0.5% for AlbAL. A volume of 250 μL of the reaction mixture was transferred into a quartz cuvette with 1 mm path length. Six far-UV spectra were accumulated in a wavelength range of 195–300 nm (203–300 nm for AlbAL) at three different temperatures (10, 21, and 90 °C) with a scanning speed of 50 nm s^−1^. For evaluation of the spectra the measured buffer blank (50 mM sodium hydrogen phosphate and 100 mM NaCl, pH 6.8) was subtracted from the averaged spectra. The temperature curves were recorded from 10–90 °C in 0.5 °C min^−1^ steps while monitoring the ellipticity at 215 nm (218 nm for ALbAL). The resulting values were recalculated into molar ellipticity [Θ]_MRW_ with consideration of concentration, path length, number of amino acids, and molecular weight of the sample. For visualization purposes, the temperature curves of AlbAS–albicidin and AlbAS–compound **5** in Fig. 3b, c were scaled to that of AlbAS using scaling factors of 1.175 and 1.091, respectively. The melting temperatures of AlbAS, AlbAS–albicidin, AlbAS–compound **5**, AlbAS–compound **7**, AlbAL, and AlbAL–albicidin were determined by nonlinear regression of the measured data using Eq. (1). In case of AlbAS–albicidin a biphasic unfolding curve was observed, which we ascribed to the two-domain arrangement. The *T*_m_ was thus determined by fitting the second transition representing the stabilized domain/species. In all other cases, the protein unfolding curves appeared as monophasic.

1 $$\Theta \left( T \right) = \frac{{\left( {\Theta _{\mathrm{N}} + m_{\mathrm{N}}T} \right) + \left( {\Theta _{\mathrm{U}} + m_{\mathrm{U}}T} \right)\mathrm{e}^{ - \frac{{{\mathrm{\Delta }}H_{\mathrm{m}}^{{\mathrm{v.H}}}}}{{RT}}\left( {\frac{{\left( {T - T_{\mathrm{m}}} \right)}}{{T_{\mathrm{m}}}}} \right)}}}{{1 + \mathrm{e}^{ - \frac{{{\mathrm{\Delta }}H_{\mathrm{m}}^{{\mathrm{v.H}}}}}{{RT}}\left( {\frac{{\left( {T - T_{\mathrm{m}}} \right)}}{{T_{\mathrm{m}}}}} \right)}}}$$

with Θ being the measured ellipticity, ${\mathrm{\Delta }}H_{\mathrm{m}}^{{{\mathrm{v.H}}}}$ denoting the van’t Hoff enthalpy at the transition point, Θ_N_ and *m*_N_ as well as Θ_U_ and *m*_U_ defining the pre-transition and post-transition phase.

### NMR spectroscopy

For binding studies with albicidin and its derivatives, each ligand was dissolved in 100% d6-DMSO and added to ^15^N-labeled AlbAS or ^2^H, ^15^N-labeled AlbAL in 50 mM sodium hydrogen phosphate (pH 6.8), 150 mM NaCl buffer (usually 0.24 mM AlbAS or AlbAL in 85% buffer, 5% d6-DMSO, and 10% D_2_O) yielding a 1.5-fold excess of the ligand. After incubation for 20 min at room temperature in the dark, the sample was centrifuged at 20 000 × *g* for 3 min and the supernatant was transferred to a 5 mm NMR tube. The AlbAS blank sample (Supplementary Figure 14) was measured in 100% buffer without addition of ligands and DMSO. Spectra were recorded on a Bruker Avance III 700 MHz NMR spectrometer (Bruker BioSpin, Rheinstetten, Germany) with a TXI probe head. Standard Bruker pulse sequences were used. 2D ^1^H-^15^N HSQC spectra were recorded at 298 K with acquisition times of 90 and 22 ms for the ^1^H and ^15^N dimension, respectively. The experiments were accumulated with ≥16 scans depending on protein sample concentration. 2D ^1^H-^15^N TROSY spectra were accumulated with 256 scans and acquisition times of 90 and 22 ms for the ^1^H and ^15^N dimension, respectively. Several AlbAS–albicidin samples were measured with the same result and a representative spectrum is given for the figures in the main manuscript and the SI. NMR experiments with AlbAS and albicidin analogs were conducted once with accumulation of ≥16 scans depending on protein concentration. For alignment of HSQC and TROSY spectra, the TROSY spectrum was corrected by 1/2 ^1^*J*_NH_ in both dimensions.

AlbAS–albicidin titration experiments were performed by means of ^1^H-^15^N HSQC experiments. Samples were prepared similarly as in the method described above: spectra were recorded at 303 K for AlbAS in buffer (50 mM sodium hydrogen phosphate and 150 mM NaCl, pH 6.8) at different protein-albicidin ratios. Albicidin was added to a 0.8 mM ^15^N-labeled AlbAS sample to the following final protein-albicidin ratios: 0.1, 0.2, 0.5, 0.8, and 1. During each titration step, the DMSO concentration was increased and reached a final concentration of 7.3% (v/v). The experiment was conducted once with accumulation of 16 scans for each titration step. The spectra were recorded with acquisition times of 90 and 20 ms in the direct ^1^H and indirect ^15^N dimension, respectively.

For the competition experiment with the DNA gyrase construct GyrBA59, 1.7 mM ATP was incubated with 3.4 mM MgCl_2_ for 10 min. Subsequently, 30 µM GyrBA59 and 3 µg supercoiled pBR322 plasmid DNA (Inspiralis) were added and incubated for another 10 min. Finally, 30 µM ^15^N-labeled AlbAS were added to the mixture followed by 30 µM albicidin (10% D_2_O and 5% DMSO final concentration). Owing to the low protein concentrations (to achieve a 1:1 ratio of gyrase and AlbAS), a ^1^H-^15^N band-selective optimized flip-angle short-transient heteronuclear multiple quantum coherence (SOFAST HMQC)^44^ experiment was performed. The ^1^H-^15^N SOFAST HMQC was recorded with 2048 scans and with acquisition times of 45 and 38 ms for the ^1^H and ^15^N dimensions, respectively. Measurements were performed at 303.2 K. The control experiment was performed under the same conditions but without the addition of MgCl_2_, ATP, pBR322 plasmid DNA, and GyrBA59.

For preliminary resonance assignments, three-dimensional HNCA and HNCO experiments were performed employing ^13^C, ^15^N-AlbAS (0.8 mM) saturated with albicidin. All NMR data were processed and analyzed with Bruker TopSpin 3.1 Software and SPARKY^45^.

### Size-exclusion chromatography with multi-angle light scattering

SEC-MALS experiments were performed at 18 °C in buffer containing 50 mM Tris (pH 7.5), 100 mM NaCl, and 0.02% NaN_3_. A sample of 225 µg of AlbAS or AlbAL (with and without saturating amounts of albicidin) were loaded onto Superdex 75 or Superdex 200 increase 10/300 columns (GE Healthcare) that were coupled to a miniDAWN TREOS three-angle light-scattering detector (Wyatt Technology) in combination with a RefractoMax520 refractive index detector. For calculation of the molecular mass, protein concentrations were determined from the differential refractive index with a specific refractive index increment (d*n*/d*c*) of 0.185 mL g^−1^. Data were analyzed with the ASTRA 6.1.4.25 software (Wyatt Technology).

### Fluorescence assay

Fluorescence quenching measurements were performed by monitoring tryptophan fluorescence of AlbAS (20 nM), AlbAL (20 nM), AlbAS_NTD_, and AlbAS_CTD_ (100 nM) on a FluoroMax 2 spectrometer from Horiba (Potsdam, Germany). The excitation wavelength was set to *λ*_exc_ = 280 nm. Emission spectra were recorded from *λ*_em_ = 290–450 nm with a scanning speed of 1 nm s^−1^ and an integration time of 1 s. The excitation and emission slit widths were set to 8 and 4 nm, respectively. Proteins were solved in 50 mM sodium phosphate buffer with 150 mM NaCl and 0.01% DMSO at pH 6.8. All measurements were performed twice and the standard deviation is given for the *K*_d_ and the Hill factor *n*. Data evaluation was performed by integration of the emission band (318–410 nm for AlbAS, 316–412 nm for AlbAL, and 319–390 nm for AlbAS_NTD_ and AlbAS_CTD_) for every titration step. The area under the curve (AUC) of the buffer blank (buffer with 0.01% DMSO) was subtracted from the measured titration data. The quenching factor was determined by subtraction of AUCs for each titration step from the AUC of the protein in the absence of ligand. For determination of the AlbAS– and AlbAL–albicidin *K*_d_ values, the data were fitted to Eq. (2).

2 $$Y = B_{{\mathrm{max}}}\frac{{[L]^n}}{{K_{\mathrm{d}}^n + [L]^n}}$$

where [*L*], *n*, and *B*_max_ denote the total ligand concentration, the Hill coefficient, and the maximum binding capacity of the protein, respectively. For the determination of the *K*_d_ for AlbAS_NTD_–albicidin and AlbAS_CTD_–albicidin as well as for AlbAS–compound **5**, the Hill coefficient was constrained to *n* = 1 for the nonlinear regression as the binding curve showed no cooperativity.

For the determination of the *K*_d_ for AlbAS–compound **7** the data were fitted to Eq. (3).

3 $$Y = \frac{{\left( {\left[ {P_{\mathrm{t}}} \right] + \left[ L \right] + K_{\mathrm{d}}} \right) - \sqrt {\left( {\left[ {P_{\mathrm{t}}} \right] + \left[ L \right] + K_{\mathrm{d}}} \right)^2 - 4\left[ {P_{\mathrm{t}}} \right]\left[ L \right]} }}{2}$$

where *P*_t_ is the total protein concentration.

### Crystallization

Crystals of unlabeled and selenomethionine-labeled AlbAS (1.4 mM AlbAS saturated with 2.5-fold albicidin in 50 mM Tris (pH 7.5), 100 mM NaCl, 4 mM DTT, and 5% DMSO) were obtained by the sitting-drop vapor-diffusion method at 18 °C with a reservoir solution composed of 0.1 M Tris/HCl (pH 8.5) and 2.0 M ammonium sulfate. For crystallization 96-well MRC plates were used. The volume of the reservoir solution was 80 μL and the crystallization drop contained 0.5 μL reservoir solution and 0.5 μL protein solution. Crystals had dimensions of 200 μm × 150 μm × 50 μm and appeared within 3 days. Crystals were cryo-protected with a solution composed of equal volumes of mother liquor and 3.4 M sodium malonate at pH 7.0 and subsequently flash-cooled in liquid nitrogen.

### Structure determination and refinement

Synchrotron diffraction data were collected at the beamline 14.1 and 14.2 of the MX Joint Berlin laboratory at BESSY (Berlin, Germany) at a wavelength of 0.97949 Å and temperature of 100 K. Diffraction data were processed with XDS^46^. Experimental phases were determined by single anomalous dispersion with the AUTOSOL routine in PHENIX^47^ using PHASER^48^ and SOLVE/RESOLVE^49^ exploiting the selenomethionine-labeled AlbAS (Supplementary Table 1). There were 35 heavy-atom sites in the asymmetric unit (FOM: 0.413, BAYES-CC: 60.3). An initial model of AlbAS was built with the program AUTOSOL in PHENIX^47^. All dihedral angles were found in favored (99.8%) and allowed (0.2%) regions of the Ramachandran plot. Our structure contains no Ramachandran plot outliers. The structure was refined by maximum-likelihood restrained refinement using in PHENIX, including TLS refinement^50^. Model adjustment and water picking was performed with COOT^51^. Geometrical restraints used in the refinement of albicidin were generated by using the Grade Web Server (http://grade.globalphasing.org) that queries the Cambridge Structural Databank with optional quantum chemical regularization. Model quality was evaluated with MolProbity^52^ and the JCSG validation server^53^. Secondary structure elements were assigned with DSSP^54^, and ALSCRIPT^55^ was used for secondary structure-based sequence alignments. Figures were prepared using PyMOL^56^ and channels were calculated with CAVER^57^.

### Data availability

The atomic coordinates and structure factor amplitudes have been deposited in the Protein Data Bank under the accession code 6ET8. Other data are available from the corresponding author upon reasonable request.

## Electronic supplementary material

Supplementary Information
