# Supplementary material for: Molecular insights into antibiotic resistance - how a binding protein traps albicidin
Source: Nat Commun. 2018 Aug 6;9:3095. doi: 10.1038/s41467-018-05551-4 (PMC6078987; doi:10.1038/s41467-018-05551-4)
Supplement: Supplementary file 1 — Supplementary Information [file 41467_2018_5551_MOESM1_ESM.pdf]

Supplementary information

for

Molecular insights into antibiotic resistance - how a binding protein traps albicidin

by

Rostock and Driller et al.

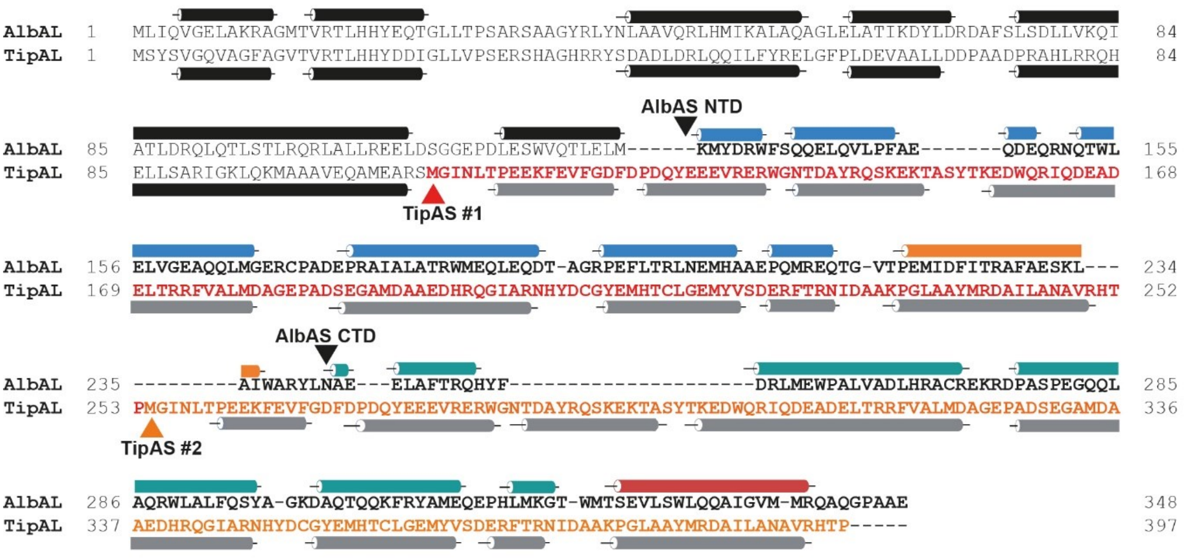

Supplementary Figure 1 Sequence alignment of AlbAL with TipAL The TipAL sequence was artificially duplicated (red and orange bold letters) in order to match the repeat of drug-binding domains in AlbAS (black, bold letters). Regions corresponding to the N-terminal HTH DNA-binding domains are shown in plain letters. Start positions of AlbAS-NTD and AlbAS-CTD are indicated by arrows above and below the alignment, respectively. α-helices are indicated by cylinders above and below the respective amino acid sequence (black cylinders: HTH domain, blue: AlbAS NTD, orange: α6, cyan: AlbAS CTD, red: α6’and grey: TipAS).

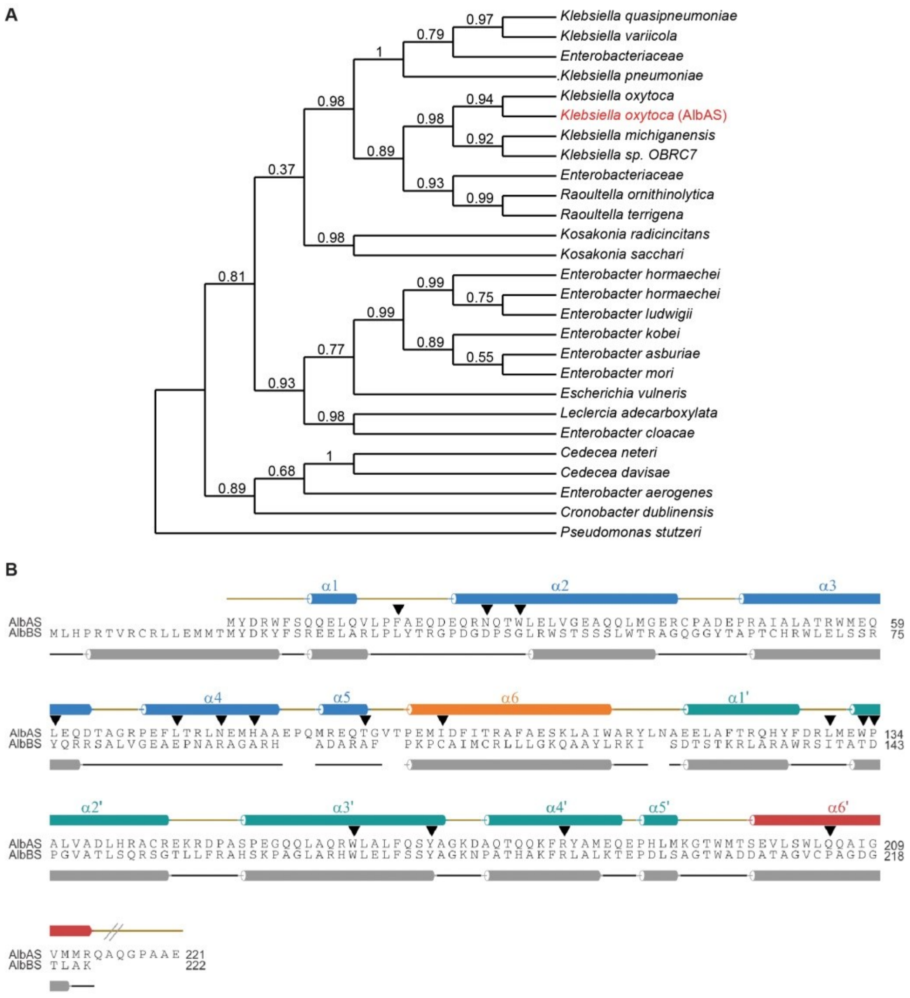

Supplementary Figure 2 Phylogenetic relationship of AlbAS and sequence alignment of AlbAS and AlbBS. A Phylogenetic relationship of AlbAS proteins based on a BLAST search with *Klebsiella oxytoca* AlbAS (red). **B** Sequence alignment of AlbAS and AlbBS. AlbAS secondary structure elements as seen in the AlbAS-albicin co-crystal structure are shown above the alignment (NTD - blue, α6 – orange, CTD – cyan, α6' - red). Predicted α-helical secondary structure elements of AlbBS are shown as cylinders below the alignment. Important AlbAS-albicidin-binding residues (triangles) are highlighted.

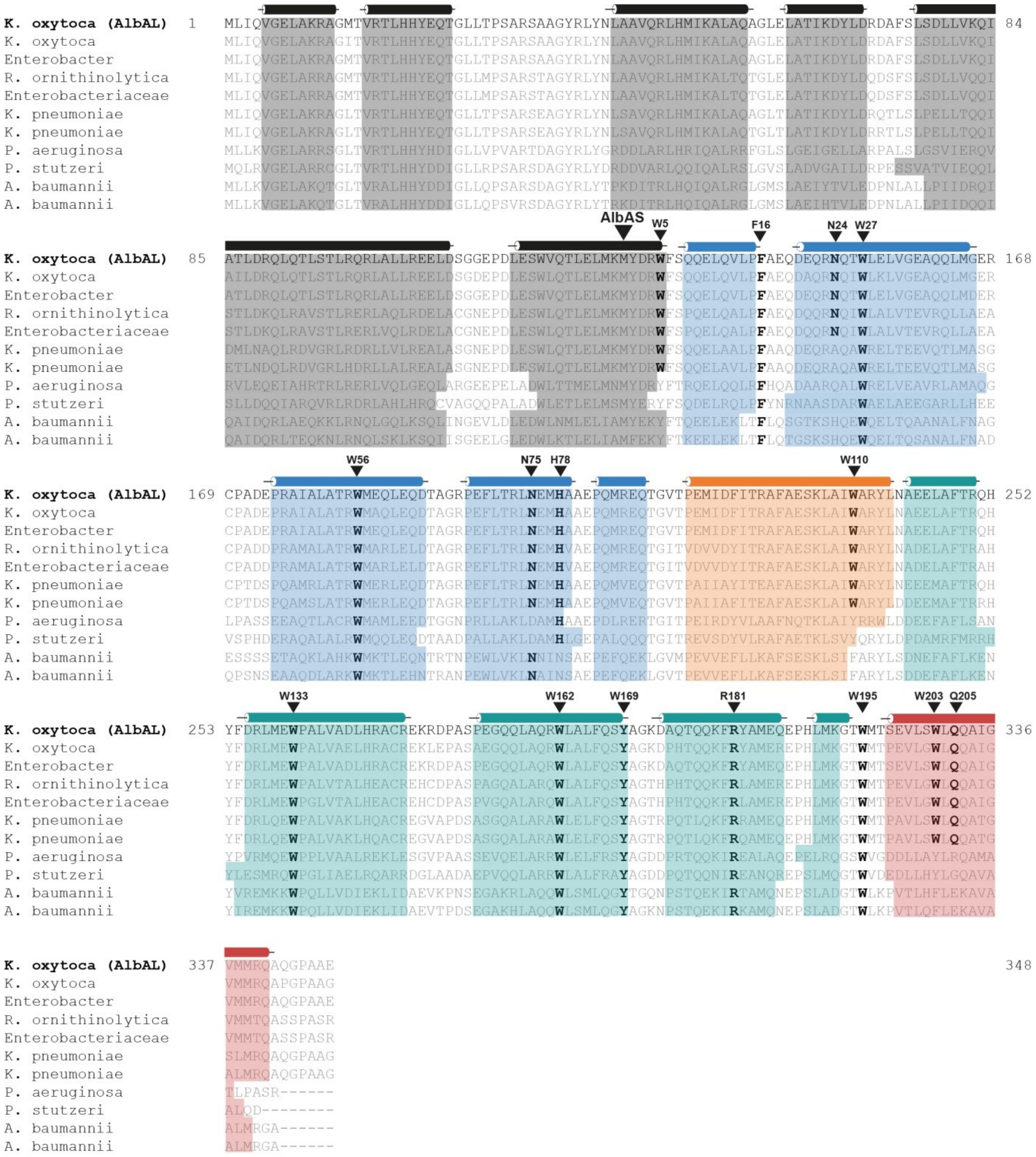

Supplementary Figure 3 Sequence alignments with secondary structure prediction of several AlbA homologs from selected ESKAPE pathogens. Predicted α-helical secondary structure elements (from PROMALS3D) are indicated by cylinders above the sequence (black cylinders: HTH domain, blue: AlbAS NTD, orange: α6, cyan: AlbAS CTD, red: α6'). Predicted α-helical secondary structure elements for the aligned MerR-family proteins are highlighted with the respective background color. Key residues are highlighted in bold letters and triangles. The percentage sequence identity for the aligned proteins to *Klebsiella oxytoca* AlbAL (100%, the protein sequence used in this work) is as followed: *Klebsiella oxytoca* 95%, *Enterobacter sp. HMSC055A11* 89%, *Raoultella ornithinolytica* 81%, *Enterobacteriaceae* 80%, *Klebsiella pneumoniae* 78% and 77%, *Pseudomonas aeruginosa* 48%, *Pseudomonas stutzeri* 47% and *Acinetobacter baumannii* both 42%.

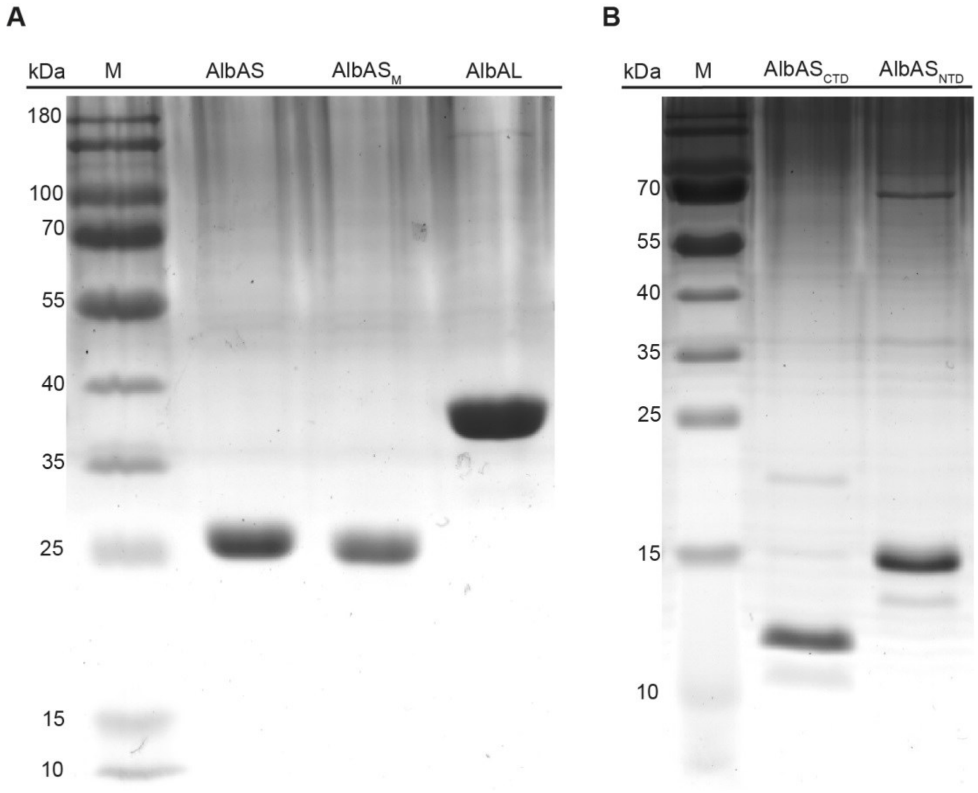

Supplementary Figure 4 Coomassie-stained SDS-PAGE of all proteins after TEV-cleavage. A AlbAS (25.9 kDa), $AlbAS_M$ after TEV-cleavage (25.7 kDa) and AlbAL (40 kDa) after TEV-cleavage. **B** $AlbAS_{CTD}$ (11.0 kDa) and $AlbAS_{NTD}$ (15.7 kDa) after TEV cleavage. M is the protein standard (10 kDa to 180 kDa).

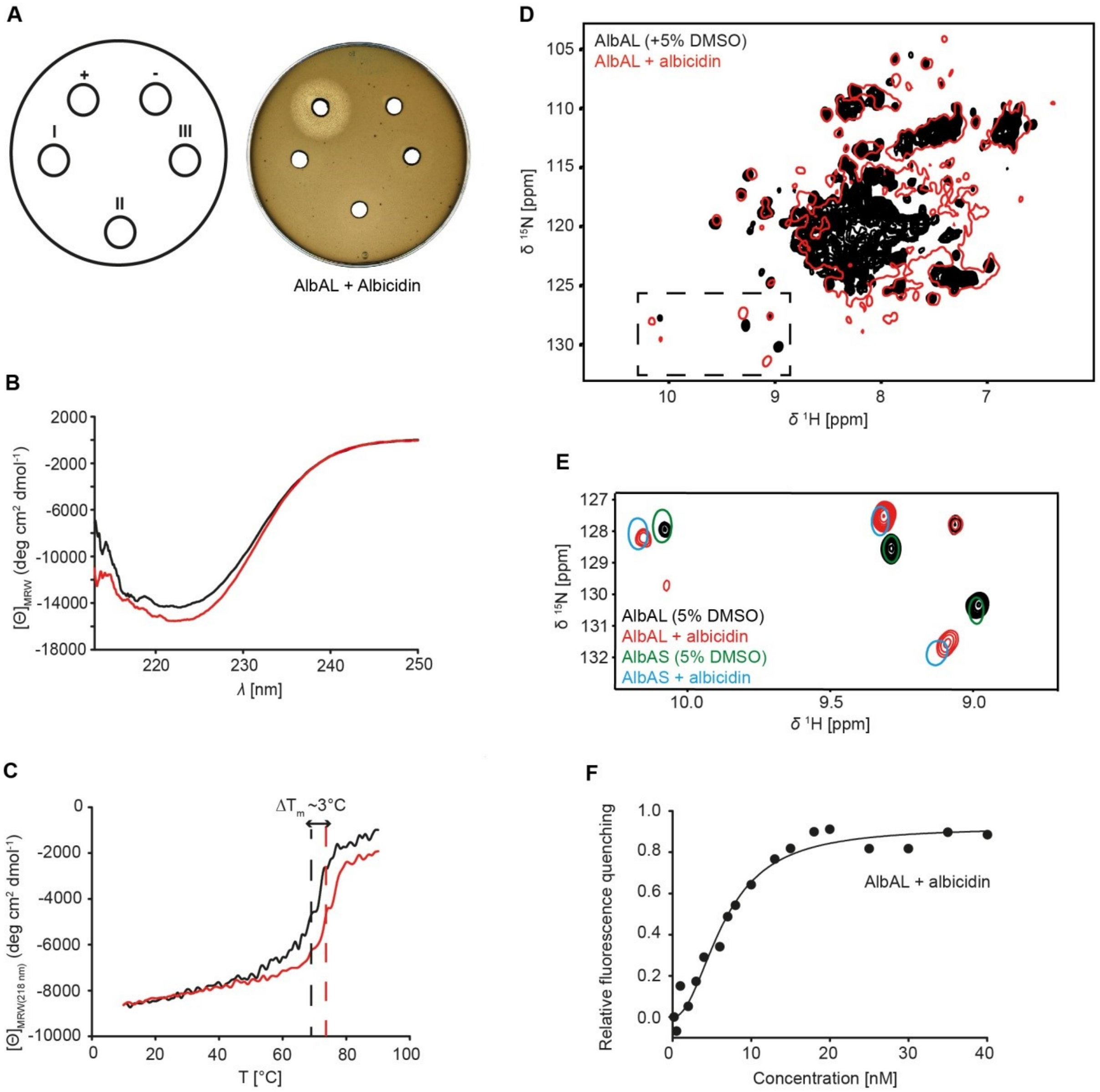

Supplementary Figure 5 Characterization of albicidin binding to AlbAL. **A** Agar diffusion assay with AlbAL (40 µM) and 40 µM albicidin (in triplicates I-III). The first plate illustrates the sample arrangement on the assay plates. Positive control with only 40 µM albicidin (+) and negative control with 40 µM protein in 5% DMSO and buffer (-) are shown on top. **B** Far-UV CD spectra of AlbAL in the absence (black) or presence (red) of albicidin (molar ratio 1.5: 1 albicidin:AlbAL) at 21 °C. **C** CD-based thermal unfolding of AlbAL in the absence (black) or presence (red) of albicidin. T_m values are indicated by dashed lines. **D** Overlay of ^{2}H, ^{15}N TROSY NMR spectra of AlbAL in the absence (black) or presence (red) of albicidin. Dashed lines highlight the reporter region enlarged in **E**. **F** Determination of binding affinities by monitoring quenching of fluorescence emission of AlbAL. Non-linear regression of fluorescence quenching data yielded a K_d of 7.4 ± 0.9 nM and a Hill coefficient, *n*, of 2.0 ± 0.23 for the interaction of albicidin with 20 nM AlbAL. The agar diffusion assay and CD experiments were conducted once. The fluorescence assay was conducted twice, and the standard deviation is given for the determined $K_{d.}$ For the NMR experiments, one purified protein sample was used and spectra accumulated with ≥ 64 scans.

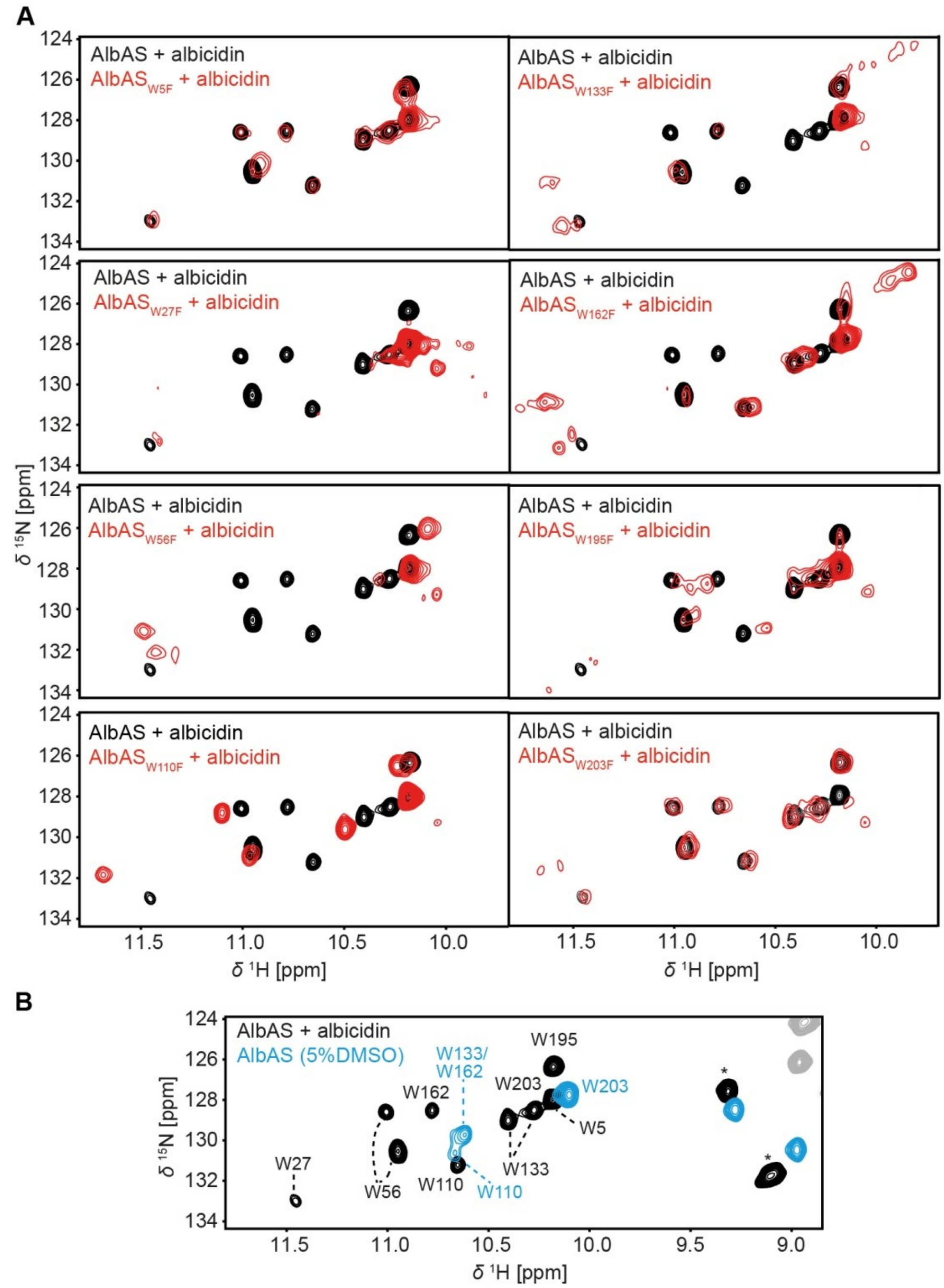

Supplementary Figure 6 Assignment of Trp side-chain signals. A Sections of ^{1}H-^{15}N HSQC spectra showing Trp Nε-Hε resonances of AlbAS (black) and the eight AlbAS Trp-to-Phe mutants (red) in the presence of albicidin. Since full backbone resonance assignments were hampered by the molecular weight and instability of AlbAS-albicidin over several days, we attempted to assign the Trp signals by generating eight single Trp-to-Phe mutants (W5F, W27F, W56F, W110F, W133F, W162F, W195F, W203F), thus anticipating the loss of a single HSQC cross peak for each mutant. The instability of the mutants, the inherent protein dynamics and the close vicinity of Trp residues in the complex usually caused the loss of more than one signal for each mutant and to non-trackable chemical shift changes, which impeded an unambiguous assignment. However, the combined information from all spectra and the knowledge of the relative positions of all Trp residues in the crystal structure helped us to tentatively assign the Trp signals as shown in **B**. The blue spectrum shows AlbAS in the absence of albicidin. Dashed lines indicate ambiguity. Signals marked with an asterisk represent backbone amides identified by 3D HNCO and HNCA experiments, whereas Trp Hε–Nε signals were further confirmed by tryptophan-selective 2D MUSIC experiments (Standard Bruker pulse sequences).

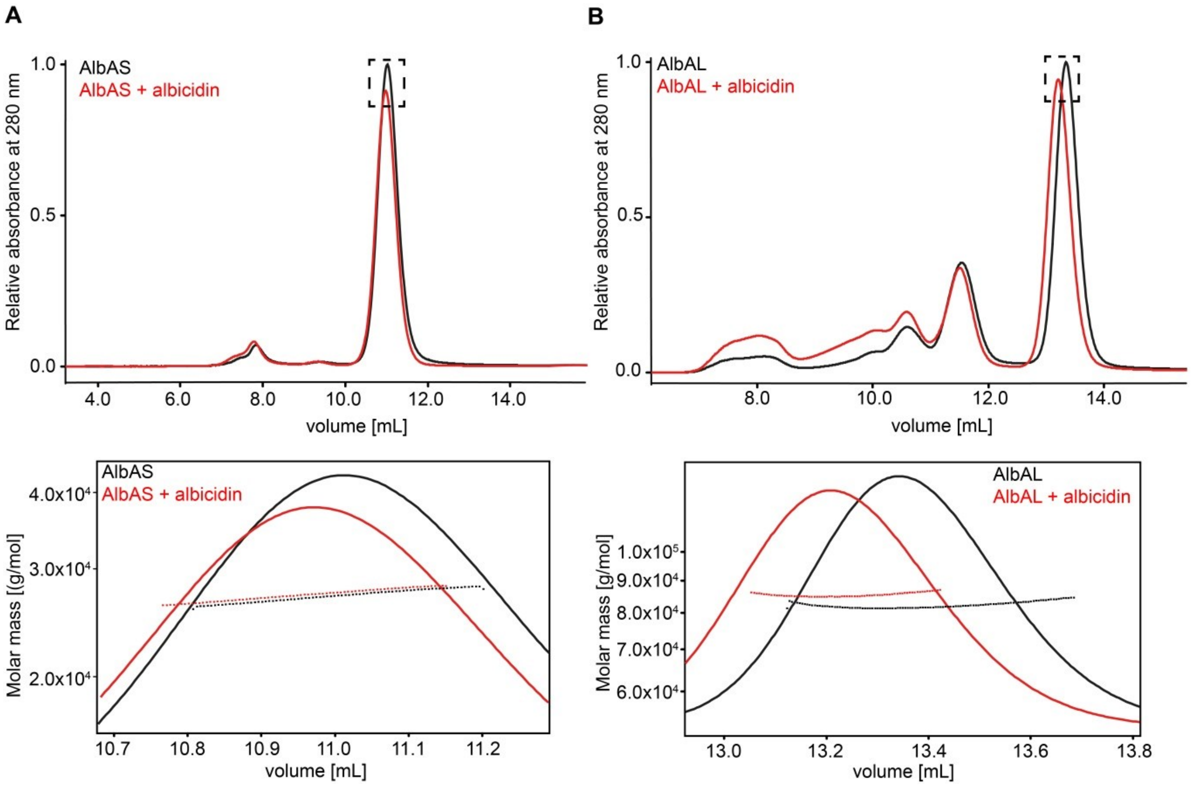

Supplementary Figure 7 SEC-MALS measurements of AlbAS, AlbAS-albicidin, AlbAL and AlbAL- albicidin to determine the molecular mass. A AlbAS and AlbAS-albicidin form monomers in solution with molecular masses of 27.1 kDa and 27.2 kDa, respectively (theoretical masses of 25.8 kDa and 26.6 kDa) **B** Analysis of AlbAL and AlbAL-albicidin showed that the proteins form dimers in solution. The doted lines correspond to the average molar mass of 82.2 kDa (black) and 85.5 kDa (red) for AlbAL and AlbAL-albicidin, respectively (theoretical masses of 40.0 kDa and 40.8 kDa). The SEC-MALS experiments were performed once.

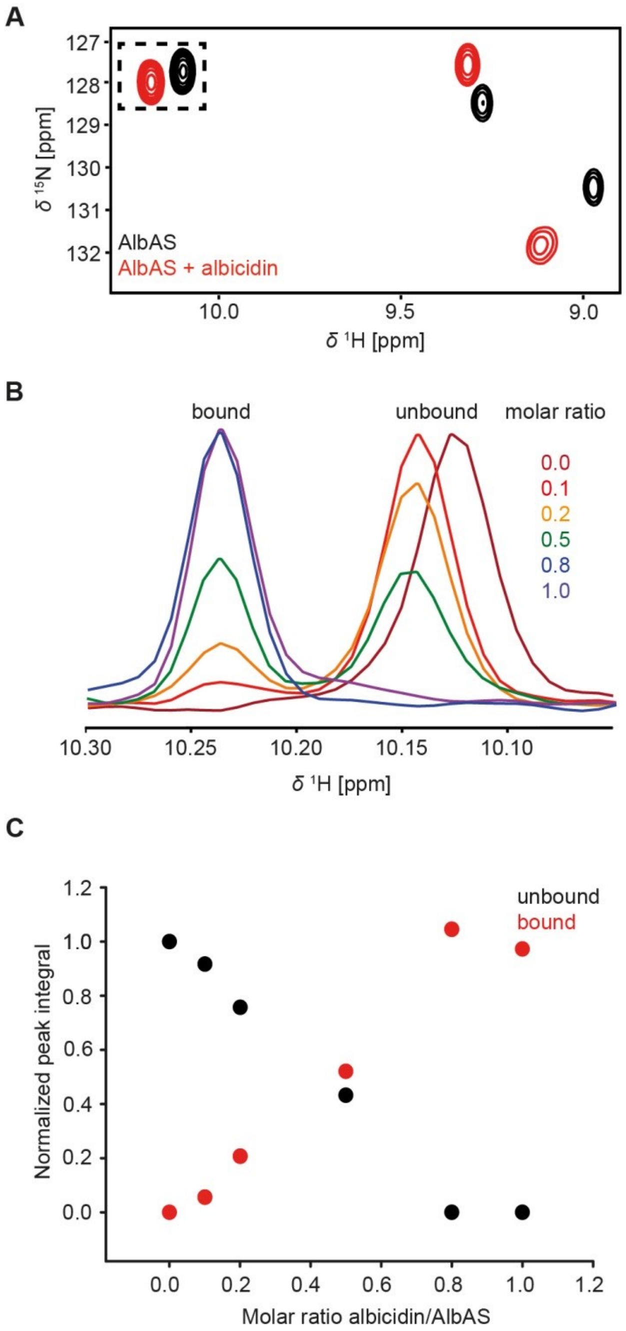

Supplementary Figure 8 NMR titration experiments. **A** Reporter region of ^{1}H-^{15}N HSQC spectra of AlbAS in the absence (black) and presence of albicidin (red) (final DMSO concentration of 5% in both cases). **B** Superposition of 1D slices extracted from ^{1}H-^{15}N HSQC spectra (dashed box in A) at different albicidin/AlbAS ratios. AlbAS without compound (0.0) contains no DMSO. **C** Plot of the normalized peak integrals of the bound (red) and unbound (black) populations as a function of the molar ratio of albicidin to AlbAS.

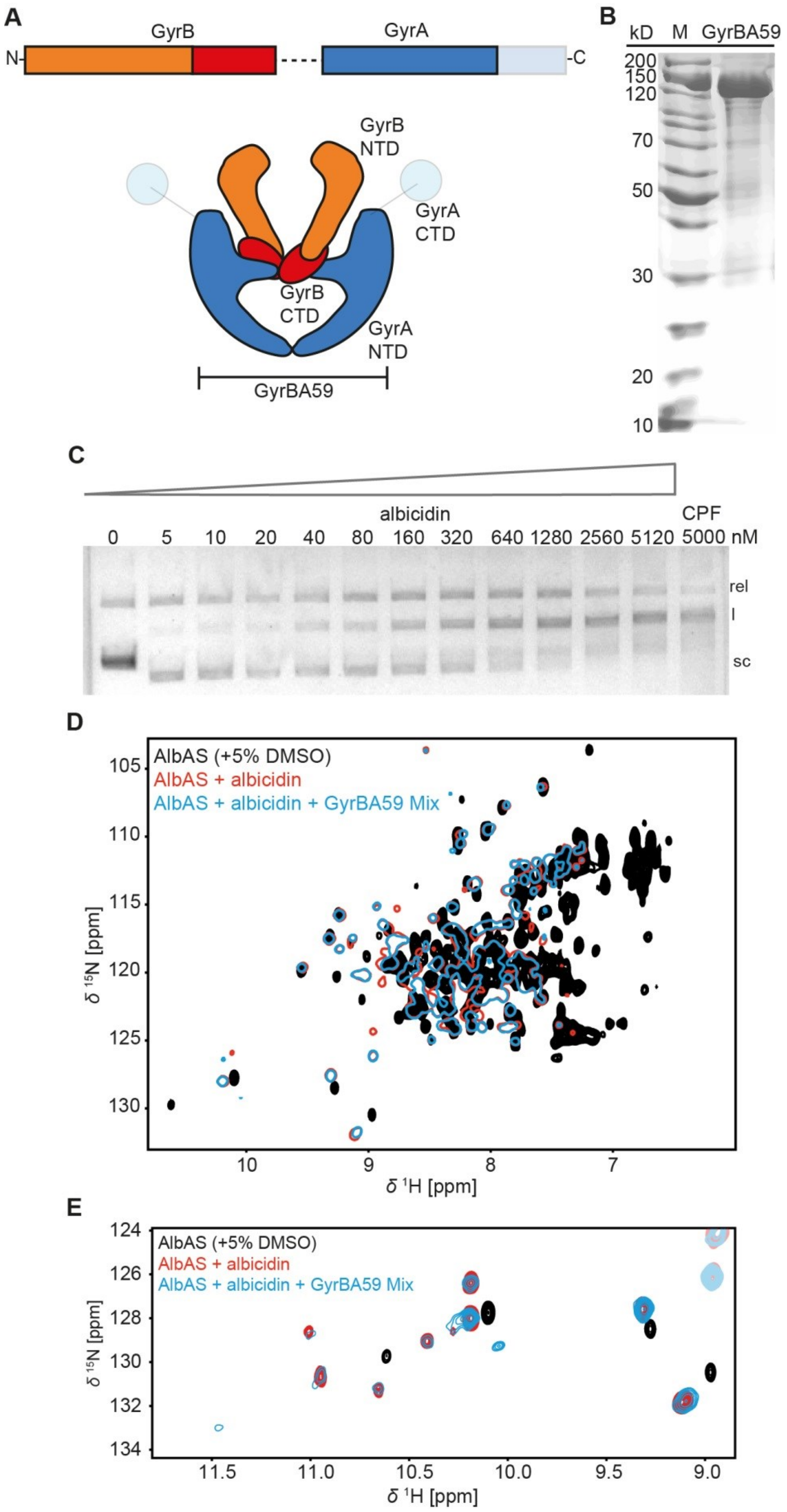

Supplementary Figure 9 Competition experiment with AlbAS and DNA gyrase construct GyrBA59. A Graphical scheme of the DNA gyrase with highlighted elements belonging to the artificial GyrBA59 fusion construct. **B** Purified GyrBA59 after TEV-cleavage (148.6 kDa) on a coomassie-stained SDS-PAGE. **C** Agarose gel-electrophoresis of the DNA-cleavage assay showing albicidin stabilizing the cleavage complex of *E.coli* GyrBA59 and pBR322 (linearized plasmid, l) over a wide range of albicidin concentrations (5 nM – 5120 nM) in presence of 1.4 mM ATP. Control reaction in presence of 5000 nM ciprofloxacin. rel: open nicked plasmid, l: linearized plasmid, sc: supercoiled plasmid. **D**

SOFAST-HMQC of ^{15}N-labelled AlbAS, purified GyrBA59 and albicidin with final concentrations of 30 µM each and addition of $MgCl_2$, ATP and pBR322 plasmid DNA. **E** Well-resolved reporter region of the SOFAST-HMQC in **D**. The NMR experiments were performed once.

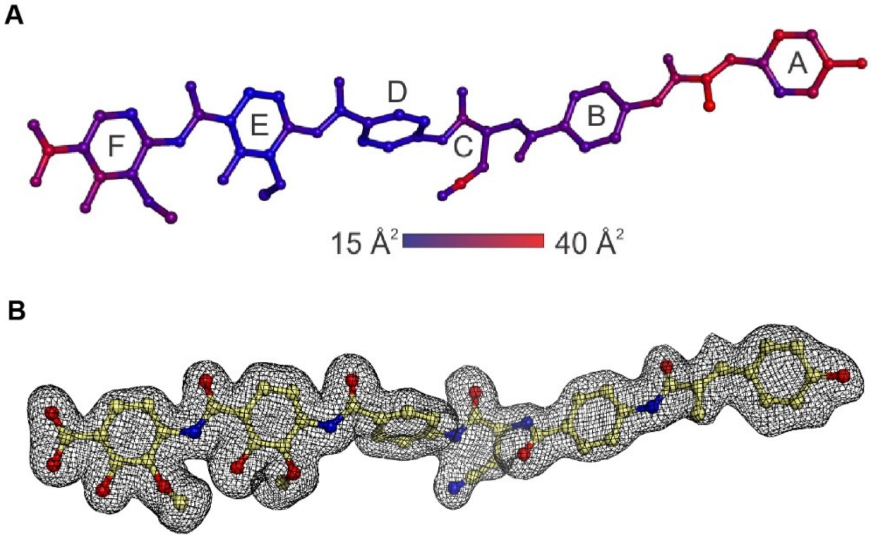

Supplementary Figure 10 Electron density and B-factor representation of albicidin. **A** AlbAS-bound albicidin with atoms colored by B-factors (low to high B-factors colored blue to red. **B** Polder electron density map of AlbAS-bound albicidin (yellow, with oxygen and nitrogen atoms colored red and blue, respectively) shown as a black mesh at a σ-level of 2.0. Orientation of albicidin is identical to Figure 5.

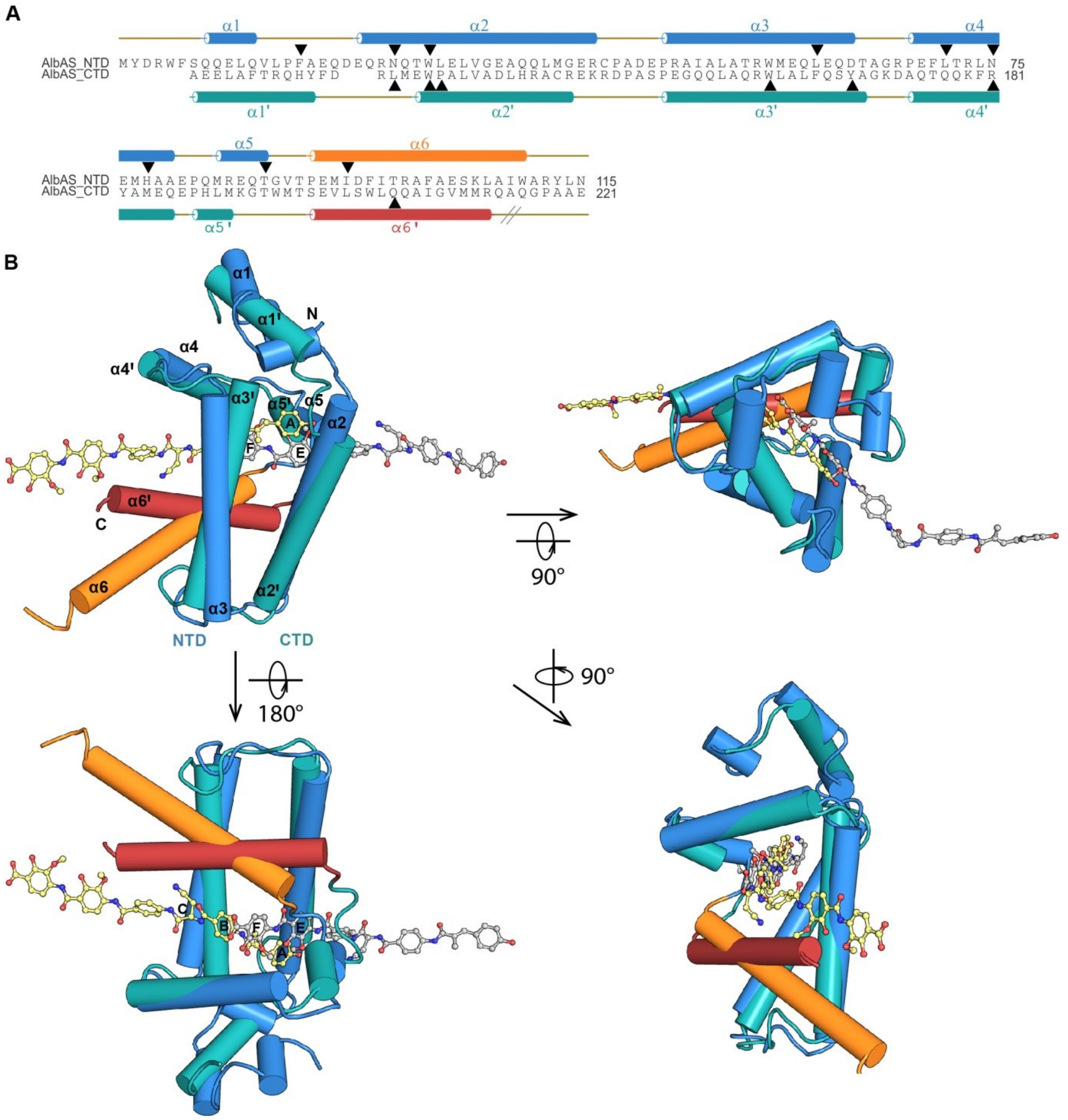

Supplementary Figure 116 Comparison of AlbAS NTD and AlbAS CTD. A Sequence alignment of the NTD (blue) and CTD (cyan). α-helical secondary structure elements as seen in the AlbAS-albicidin crystal structure are shown above and below the alignment as cylinders. Helices α6 (orange) and α6’ (red) are highlighted. Important albicidin binding residues are marked with triangles. **B** Structural alignment of AlbAS NTD (blue) and CTD (cyan) showing the binding and entrance sites of albicidin used by the two repeat units. Albicidin is shown in ball-and-stick representation and colored grey (for the NTD) and yellow (for the CTD). Helices α6 (orange) and α6’ (red) are highlighted. Different orientations are marked with arrows and rotation axis. The alignment in the left corner has the same orientation as in Figure 5B of the main text.

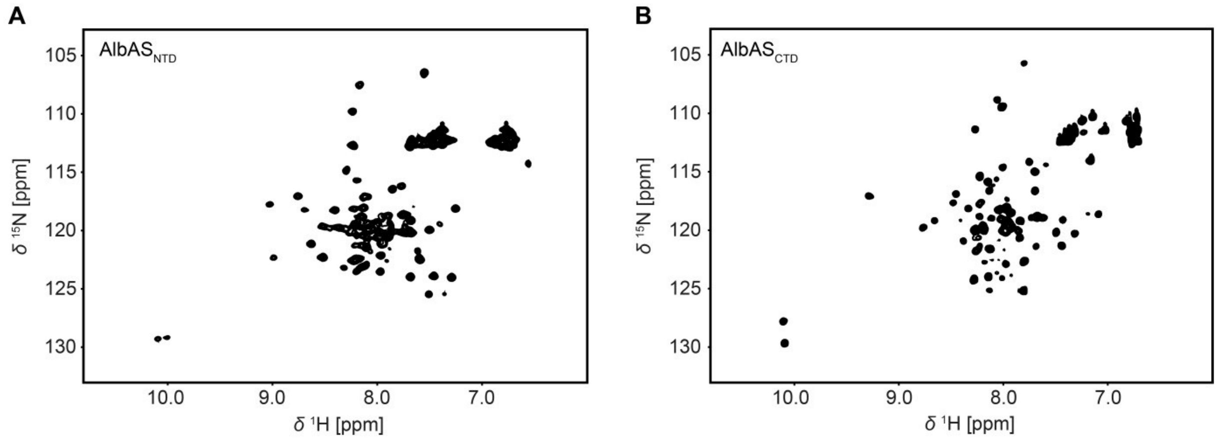

Supplementary Figure 12 ^{1}H-^{15}N HSQC spectra of truncated AlbAS variants. Spectra of **A** AlbAS$_{NTD}$ and **B** AlbAS$_{CTD}$ are shown in black. The NMR spectra were performed using one purified protein sample each and accumulated with 16 scans.

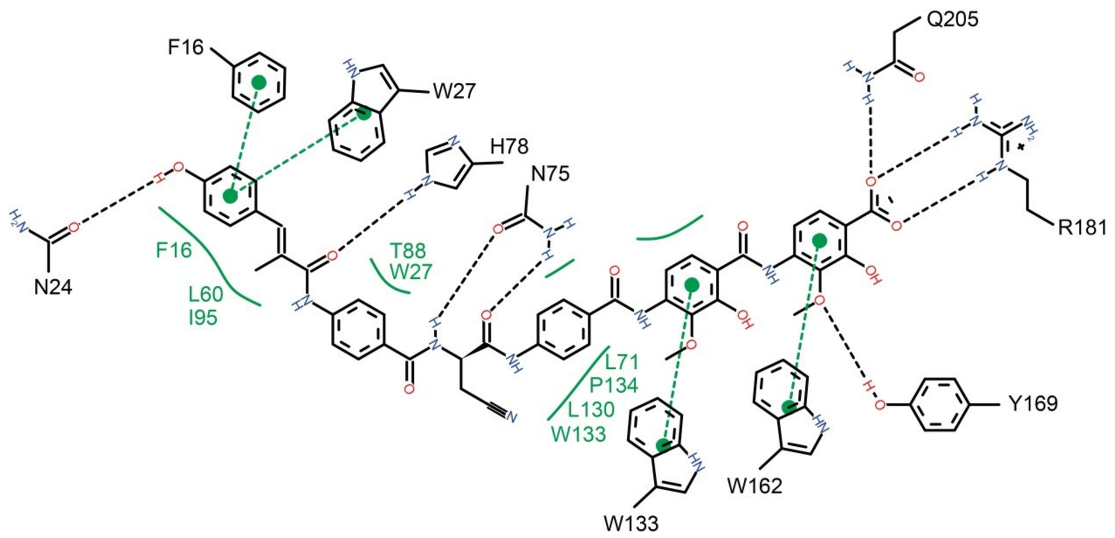

Supplementary Figure 13 Network of interactions in the albicidin binding pocket. Hydrogen bonds are illustrated as dashed lines. π-π stacking is highlighted with green dashed lines. Residues contributing to the hydrophobic binding tunnel are highlighted in green.

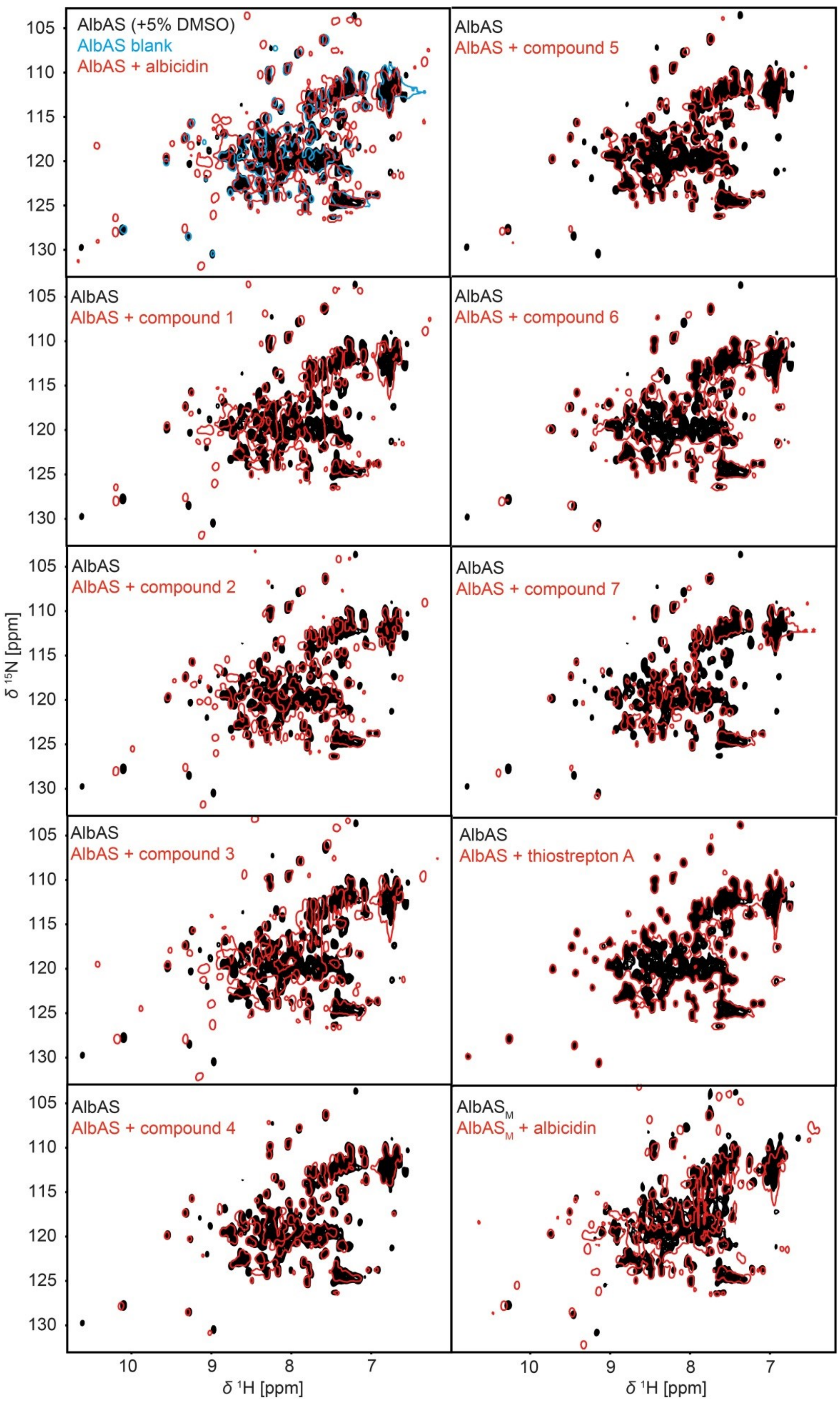

Supplementary Figure 14 Superposition of ^{1}H-^{15}N HSQC spectra of AlbAS with albicidin and different compounds. The first spectrum (same spectrum as presented in Figure 3E) shows AlbAS (black) in 95% buffer/5% DMSO, unbound- AlbAS blank in 100% buffer without compound (blue) and AlbAS with albicidin (red). The following spectra show AlbAS (black) with the corresponding titrated compound (red). AlbAS-albicidin measurements were performed on five different samples and a representative experiment is shown in the figure. The NMR measurements using albicidin derivatives were conducted once.

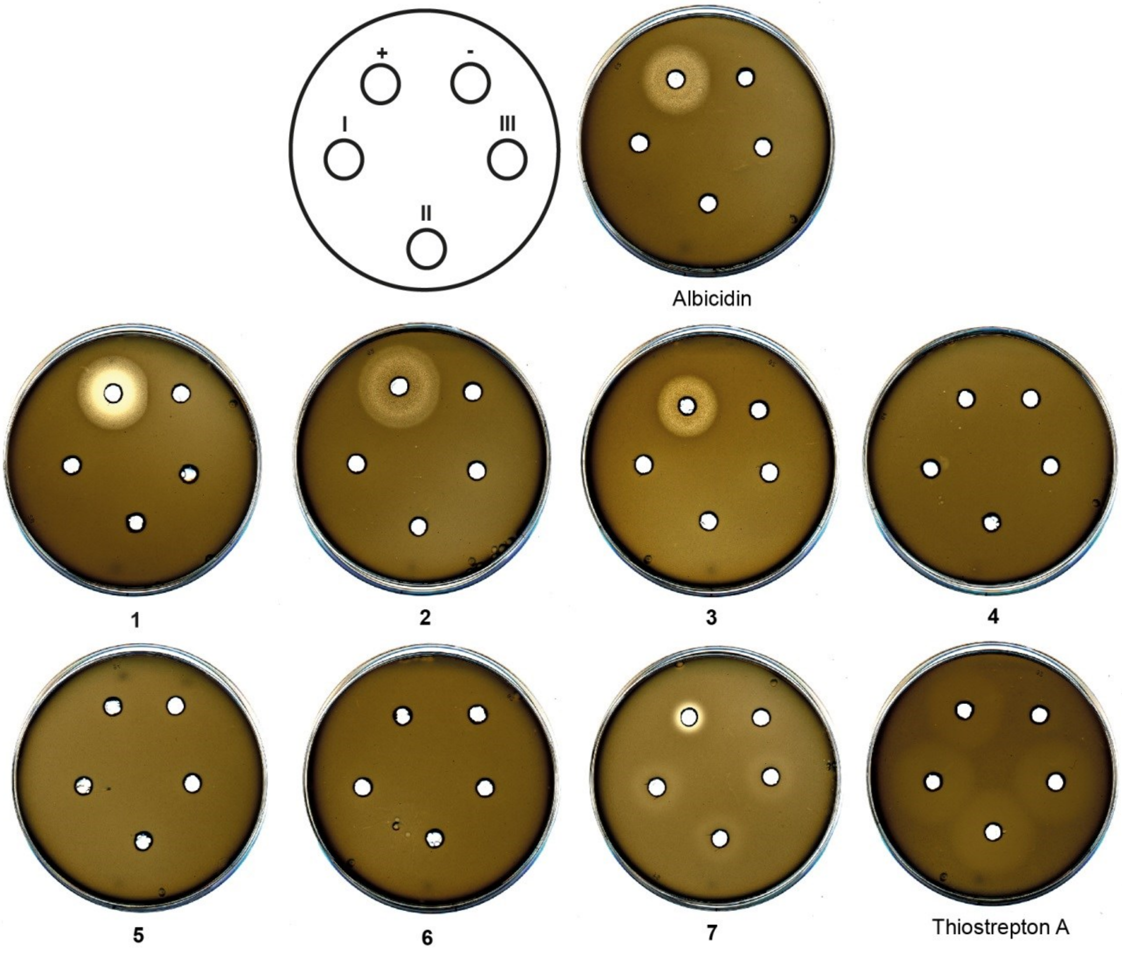

Supplementary Figure 15 Agar diffusion assay with different compounds. AlbAS (40 µM) with albicidin, thiostrepton A or compound **1**-**7** (40 µM each) in triplicates I-III. The first plate illustrates the sample arrangement on the assay plates. Positive control with only 40 µM albicidin (+) and negative control with 40 µM protein in 5% DMSO and buffer (-) are shown on top. Agar diffusion assays with AlbAS-albicidin were conducted three times and a representative result is shown in the figure. The assays with AlbAS and derivatives were performed once.

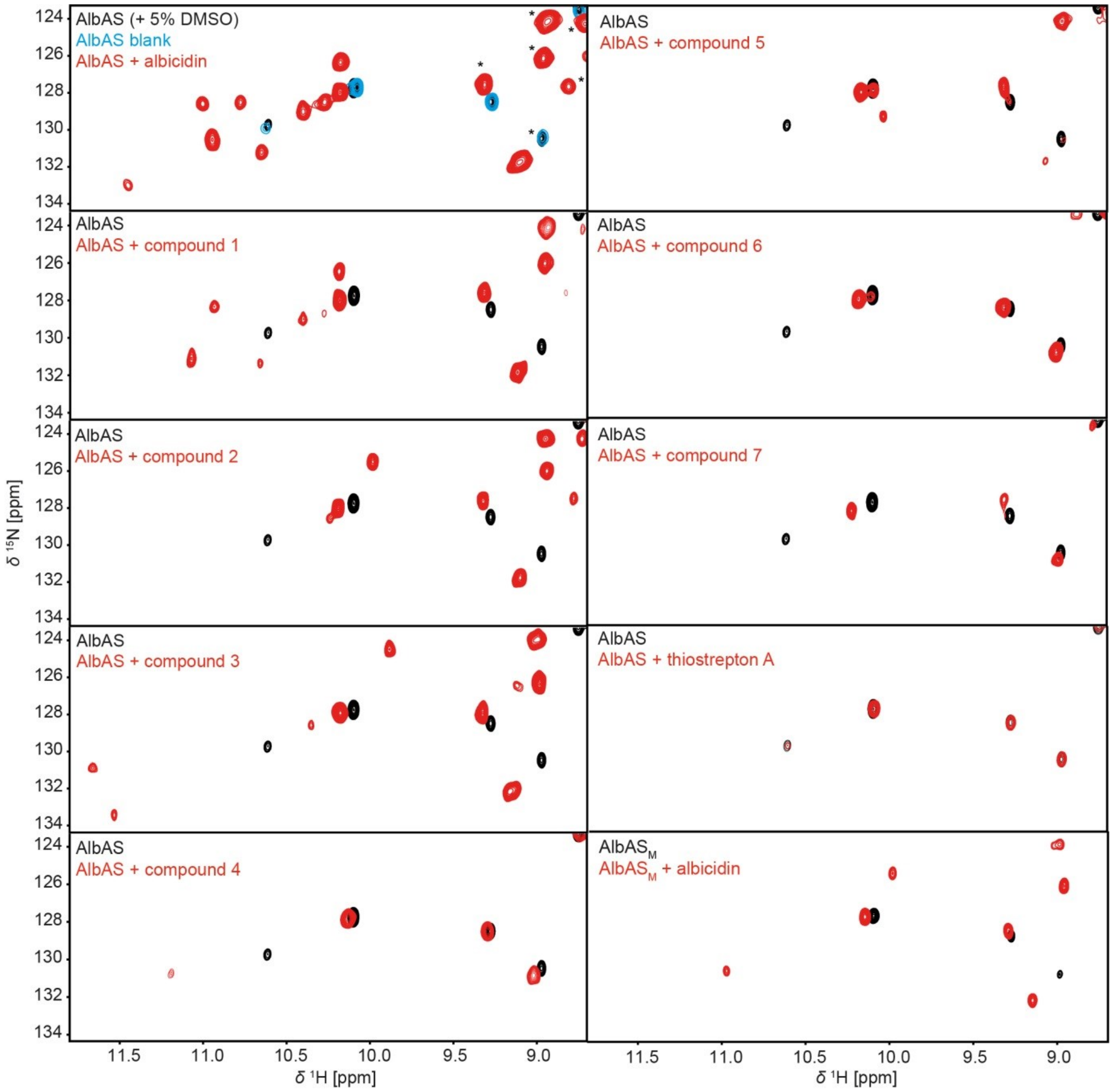

Supplementary Figure 16 Sections of ^{1}H-^{15}N HSQC downfield region of AlbAS-albicidin and derivatives. The first spectrum shows AlbAS (black) in 95% buffer/5% DMSO, unbound-AlbAS blank in 100% buffer without compound (blue) and AlbAS with albicidin (red). Signals marked with an asterisk could be assigned as backbone amides. AlbAS-albicidin measurements were conducted five times and a representative experiment is shown in the figure. The NMR measurements for AlbAS with derivatives were conducted once.

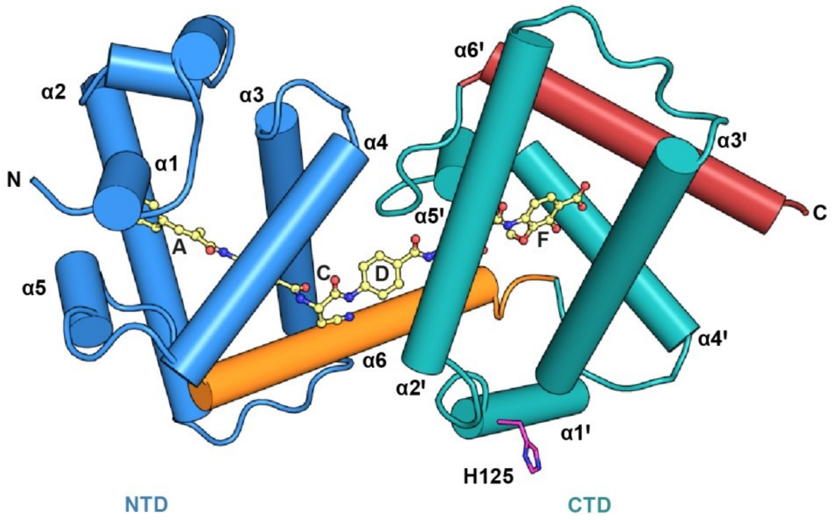

Supplementary Figure 17 Position of residue H125 on the surface of the AlbAS-albicidin complex.

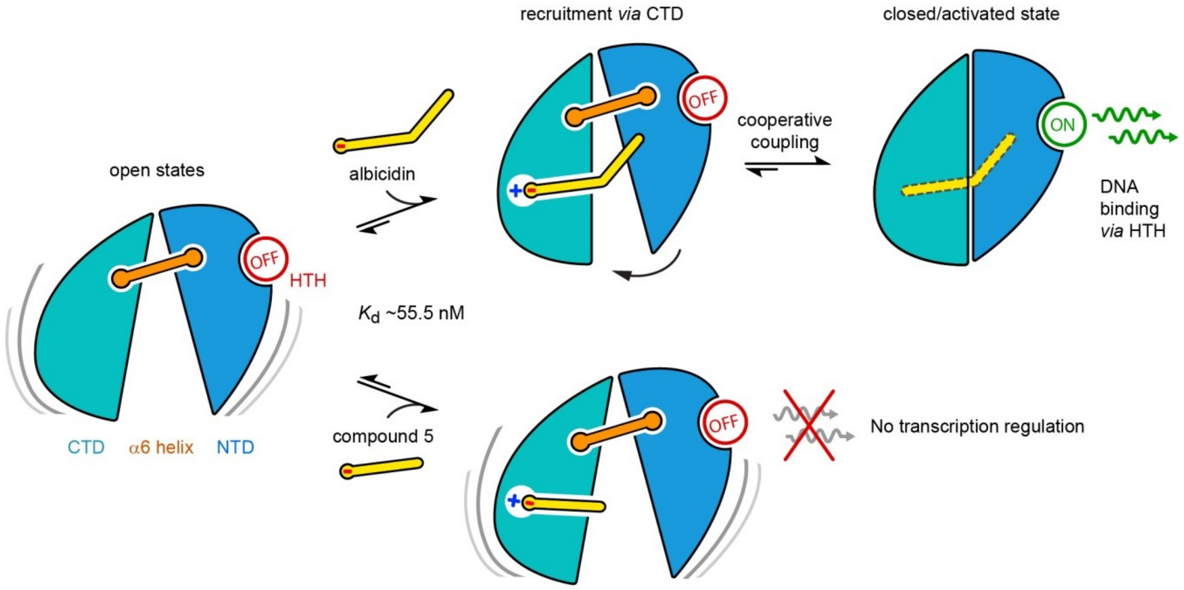

Supplementary Figure 18 Model of the binding mechanism of AlbAS. In the absence of albicidin, AlbAS undergoes inherent domain-domain motions on the micro-to-millisecond timescale. In this conformational ensemble, the N-terminal HTH domain is not competent for DNA binding. Upper lane: Initial anchoring of the C-terminal segment of albicidin is mediated via the CTD of AlbAS (the salt-bridge between R181 of AlbAS and building block F of albicidin is indicated by the charges). The subsequent binding of the NTD to the N-terminal segment of albicidin is highly cooperative giving an effective K_d for the entire binding process of ≤ 5.6 ± 0.2 nM. Only this mutual fixation of the NTD and CTD causes the HTH to adopt a DNA-binding competent state. Drug binding and transcriptional regulation of MerR-related genes are thus strongly coupled. Lower lane: C-terminal fragments of albicidin (here compound 5), which are still strongly captured by the CTD (K_d of approx. 55.5 ± 3.6 nM), do not or only inefficiently bridge the CTD and NTD. The HTH domain is thus decoupled from the drug-binding event and is not able to initiate transcription. Notably, in the case of AlbAL, these processes occur in a functional dimer capable of DNA binding after albicidin capture.

Supplementary Table 1 Data collection, phasing and refinement statistics (SAD-SeMet).

	SAD
**Data collection**	
Space group	*C*222$_1$
Cell dimensions	
a, *b*, *c* (Å)	54.07, 123.35, 159.2
a, b, g (°)	90, 90, 90
Resolution (Å)	50.00 - 1.70 (1.74 - 1.70)
R_{meas} (%)	11.9 (179.6)
I / s*I*	16.53 (1.49)
Completeness (%)	100 (100)
Redundancy	13.50 (13.62)
**Refinement**	
Resolution (Å)	50.00 - 1.70 (1.76 - 1.70)
No. reflections	58928 (5818)
R_{work} / R_{free}	0.177 / 0.204
No. atoms	
Protein	3789
Albicidin	124
Water	372
B-factors	
Protein	31.3
Albicidin	27.9
Water	35.4
R.m.s deviations	
Bond lengths (Å)	0.009
Bond angles (°)	1.02

*Number of xtals for each structure should be noted in footnote. *Values in parentheses are for highest-resolution shell.

[AU: Equations defining various *R*-values are standard and hence are no longer defined in the footnotes.]
[AU: Phasing data should be reported in Methods section.]
[AU: Ramachandran statistics should be in Methods section at end of Refinement subsection.]
[AU: Wavelength of data collection, temperature and beamline should all be in Methods section.]

Supplementary Table 2 Codon optimized DNA sequences.

Codon optimized AlbAS DNA sequence
CATATGTATGATCGTTGGTTTAGCCAGCAAGAACTGCAGGTTCTGCCGTTTGCAGAACAGGATG<br>AACAGCGTAATCAGACCTGGCTGGAACTGGTTGGTGAAGCACAGCAGCTGATGGGTGAACGTT<br>GTCCGGCAGATGAACCGCGTGCAATTGCCCTGGCAACCCGTTGGATGGAACAGCTGGAACAGG<br>ATACCGCAGGTCGTCCGGAATTTCTGACCCGTCTGAATGAAATGCATGCAGCAGAACCGCAGA<br>TGCGTGAACAGACCGGTGTTACTCCGGAAATGATTGATTTTATCACCCGTGCATTTGCCGAAAG<br>CAAACTGGCAATTTGGGCACGTTATCTGAATGCAGAAGAACTGGCATTTACCCGTCAGCATTAT<br>TTTGATCGTCTGATGGAATGGCCTGCACTGGTTGCCGATCTGCATCGTGCATGTCGTGAAAAAC<br>GTGATCCGGCAAGTCCGGAAGGTCAGCAGCTGGCACAGCGTTGGCTGGCACTGTTTCAGAGCT<br>ATGCAGGTAAAGATGCACAGACCCAGCAGAAATTTCGTTATGCAATGGAACAAGAACCGCATC<br>TGATGAAAGGCACCTGGATGACCAGCGAAGTGCTGAGCTGGCTGCAGCAGGCAATTGGTGTT<br>ATGATGCGTCAGGCACAGGGTCCGGCAGCAGAATAAGCGGCCGC

Codon optimized AlbAL DNA sequence
ATGCTGATCCAAGTGGGAGAGTTGGCGAAACGTGCGGGTATGACAGTGCGTACGCTGCATCAC<br>TACGAGCAAACCGGCCTGCTGACTCCGAGTGCGCGTTCTGCTGCTGGCTATCGCCTGTACAACC<br>TTGCAGCAGTTCAGCGTCTGCACATGATCAAAGCGTTAGCTCAGGCTGGCTTAGAGCTGGCAA<br>CCATCAAAGACTATCTGGATCGCGACGCATTTTCCTTGAGCGATCTGCTGGTGAAACAGATTGC<br>CACCTTAGATCGGCAGCTGCAGACTCTGTCGACCCTTCGCCAACGCCTGGCTCTGCTGCGCGAA<br>GAACTCGATAGTGGCGGTGAACCGGACCTGGAATCGTGGGTGCAGACCTTAGAGCTGATGAA<br>GATGTACGATCGCTGGTTTTCCCAACAGGAGCTTCAGGTCTTGCCGTTTGCCGAACAAGACGAA<br>CAGCGCAATCAGACCTGGCTCGAGCTTGTAGGCGAAGCGCAACAACTCATGGGCGAACGTTGC<br>CCTGCCGATGAACCCCGCGCGATTGCGCTGGCCACACGCTGGATGGAACAGCTGGAACAGGAT<br>ACAGCGGGACGTCCGGAATTCCTGACGCGCCTCAATGAGATGCATGCCGCTGAACCGCAAATG<br>CGTGAACAGACCGGTGTAACGCCCGAAATGATTGACTTCATCACCCGTGCGTTTGCCGAAAGCA<br>AATTAGCGATTTGGGCCCGTTATCTGAACGCGGAAGAACTGGCGTTTACGCGTCAGCACTATTT<br>CGATCGGCTGATGGAATGGCCAGCACTTGTTGCGGATTTGCATCGCGCCTGTCGGGAGAAACG<br>CGATCCAGCATCTCCGGAAGGGCAGCAATTAGCCCAACGCTGGTTGGCGCTCTTCCAGAGCTAT<br>GCCGGCAAAGACGCACAGACTCAGCAGAAGTTTCGCTACGCAATGGAACAGGAGCCGCATCTG<br>ATGAAAGGTACGTGGATGACCTCAGAAGTTCTGAGCTGGTTGCAGCAGGCCATTGGGGTCATG<br>ATGCGTCAAGCGCAAGGTCCTGCAGCTGAGTAA

Supplementary Table 3 Oligonucleotides used for cloning.

Oligonucleotide sequence (5' – 3')	Name
CTTGTATTTCCAGGGCCAATGGATCGTCTGATGGAATGGCC	AlbAS$_{CTD}$_fw
TCCACCAGTCATGCTAGCCATTTATTCTGCTGCCGGACCCTGTGCCT	AlbAS$_{CTD}$_rv
CTTGTATTTCCAGGGCCA ATGTATGATCGTTGGTTTAG	AlbAS$_{NTD}$_fw
TCCACCAGTCATGCTAGCCATTTAATTCAGATAACGTGCCCAAATTGC	AlbAS$_{NTD}$_rv
CGTATTACCGCCTTTGAGTGAGCTGATACC	Fw_all_AlbAS$_{M}$
GGTATCAGCTCACTCAAAGGCGGTAATACG	Rv_all_AlbAS$_{M}$
ATTTCTGACCCGTCTGGCAGAAATGCATGCAGCAG	AlbAS$_{M}$_N75A_fw
CAGACGGGTCAGAAATTCCGGACGACCTGCGG	AlbAS$_{M}$_N75A_rv
GACCCAGCAGAAATTTGCATATGCAATGGAACAAGAA	AlbAS$_{M}$_R181A_fw
AAATTTCTGCTGGGTCTGTGCATCTTTACCTGCATAG	AlbAS$_{M}$_R181A_rv
AGTGCTGAGCTGGCTGGCACAGGCAATTGGTGTTATG	AlbAS$_{M}$_Q205A_fw
CAGCCAGCTCAGCACTTCGCTGGTCATCCAGGTGCCT	AlbAS$_{M}$_Q205A_rv
CGTATTACCGCCTTTGAGTGAGCTGATACC	AlbAS_Trp_all_fw
GGTATCAGCTCACTCAAAGGCGGTAATACG	AlbAS_Trp_all_rv
CCATATGTATGATCGTTTTTTTAGCCAGCAAGAACTG	AlbAS_W5F_fw
ACGATCATACATATGGCCCTGGAAATACAAGTTTTCG	AlbAS_W5F_rv
GAACAGCGTAATCAGACCTTTCTGGAACTGGTTGGT	AlbAS_W27F_fw
GGTCTGATTACGCTGTTCATCCTGTTCTGCAAA	AlbAS_W27F_rv
GCCCTGGCAACCCGTTTTATGGAACAGCTGGAACAG	AlbAS_W56F_fw
ACGGGTTGCCAGGGCAATTGCACGCGGTTCATC	AlbAS_W56F_rv
GAAAGCAAACTGGCAATTTTTGCACGTTATCTGAATGCA	AlbAS_W110F_fw
AATTGCCAGTTTGCTTTCGGCAAATGCACGGGTGAT	AlbAS_W110F_rv
TTTGATCGTCTGATGGAATTTCCTGCACTGGTTGCCGAT	AlbAS_W133F_fw
TTCCATCAGACGATCAAAATAATGCTGACGGGTAAA	AlbAS_W133F_rv
AGCAGCTGGCACAGCGTTTTCTGGCACTGTTTCAGAGC	AlbAS_W162F_fw
ACGCTGTGCCAGCTGCTGACCTTCCGGACTTGC	AlbAS_W162F_rv
CCGCATCTGATGAAAGGCACCTTTATGACCAGCGAAGTGCTG	AlbAS_W195F_fw
GGTGCCTTTCATCAGATGCGGTTCTTGTTCCATTGCATA	AlbAS_W195F_rv
ACCAGCGAAGTGCTGAGCTTTCTGCAGCAGGCAATTGGT	AlbAS_W203F_fw
GCTCAGCACTTCGCTGGTCATCCAGGTGCCTTTCAT	AlbAS_W203F_rv
TAATACGACTCACTATAGGG	Sequencing_T7
TGCTAGTTATTGCTCAGCGG	Sequencing_T7-term
AACTTGTATTTCCAGGGCATGCTGATTCAGGTTGGT	pET28a_AlbAL_TEV_fw
GCTTCCTTTCGGGCTTTGTTATTCTGCGGCAGGACCCT	pET28a_AlbAL_TEV_rv
TGGATCCCATATGTCGAATTCTTATGACT	pET28a_GyrBA59_fw
GGTGCTCGAGGTTAGGCGGTGATTTCAGTACGA	pET28a_GyrBA59_rv
GCGAATATCGATATTAGCGACCTTGCG	GyrBA59_fusion_fw
CTCTCGCAAGGTCGCTAATATCGATATT	GyrBA59_fusion_rv

Supplementary methods 1 Chemical synthesis and analytical data of compound 2.

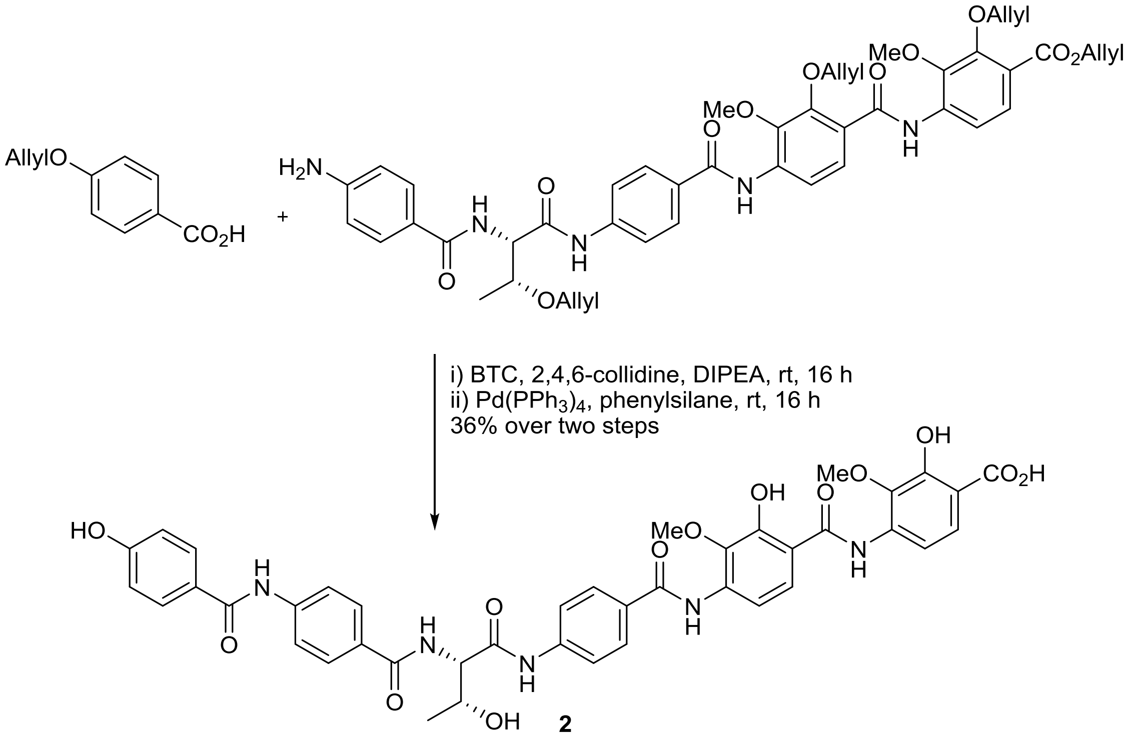

Synthesis of compound **2**:

To a solution of commercially available 4-(allyloxy)-benzoic acid (1.5 eq, 16 mg, 88 µmol), and triphosgene (0.6 eq, 11 mg, 35 µmol), dissolved in anhydrous THF (3 mL), was slowly added 2,4,6-collidine (8 eq, 62 µL, 0.47 mmol) via syringe. After 5 minutes of stirring, a solution of the corresponding allyl-protected aniline pentapeptide9 (1 eq, 50 mg, 59 µmol) and DIPEA (10 eq, 100 µL, 590 µmol), dissolved in anhydrous THF (2 mL), was added. After 16 h stirring at room temperature, the reaction was quenched by the addition of ice. The mixture was extracted with dichloromethane (3 x 20 mL). The combined organic extracts were washed with 1 N HCl (aq) (3 x 20 mL), sat. NaHCO3 (3 x 20 mL) and brine (1 x 20 mL), dried over Na2SO4, filtered and evaporated to dryness. The residue was dissolved in anhydrous THF (5 mL), and subsequently phenylsilane (8 eq, 58 µL, 0.47 mmol) and tetrakis(triphenylphosphine)palladium(0) (0.4 eq, 27 mg, 24 µmol) were added. The reaction was stirred for 16 h at room temperature under exclusion of light. The mixture was clarified via filtration through a 0.2 µm PTFE syringe filter and directly applied to a preparative RP-HPLC purification yielding compound 2 (17 mg, 21 µmol) as a white fluffy powder.

Analytical data for compound **2**:

^{1}H-NMR (500 MHz, DMSO-d6): δ (ppm) = 1.20 (d, *J* = 6.3 Hz, 3H), 3.78 (s, 3H), 3.91 (s, 3H), 4.15-4.24 (m, 1H), 4.59 (dd, *J* = 4.8 Hz, *J* = 8.1 Hz, 1H), 5.08 (s, 1H), 6.88 (d, *J* = 8.6 Hz, 2H), 7.56-7.62 (m, 2H),

7.80 (t, *J* = 8.5 Hz, 3H), 7.86-7.95 (m, 8H), 7.97 (d, *J* = 8.6 Hz, 2H), 8.04 (d, *J* = 8.9 Hz, 1H), 8.08 (d, *J* = 8.1 Hz, 1H), 9.66 (s, 1H), 10.16 (s, 1H), 10.22 (s, 1H), 10.37 (s, 1H), 11.16 (s, 1H), 11.53 (s, 1H).

HRMS (APCI): m/z calc. for $C_{41}H_{37}N_5O_{13}$ $[M-H]^-$: 806.2304; exp. 806.2336.
